# A Blockchain-Enabled Federated Neuro-Symbolic Framework for Secure Wearable Biosensor-Based Health Monitoring

**DOI:** 10.3390/bios16080442

**Published:** 2026-08-16

**Authors:** Khulud Salem Alshudukhi, Noshina Tariq

**Affiliations:** 1 Department of Computer Science, College of Computer and Information Sciences, Jouf University, Sakaka 72388, Al-Jouf, Saudi Arabia; 2 Department of Artificial Intelligence and Data Science, National University of Computer and Emerging Sciences, Islamabad 44000, Pakistan

**Keywords:** neuro-symbolic AI, AI in wearable biosensing, remote patient monitoring, federated learning, HomomorphicEncryption, Bio-ClinicalBERT, anomaly detection, blockchain, sharded tangle ledger, Honey Encryption, IoD

## Abstract

Wearable biosensors generate continuous physiological data in smart Internet of Disease (IoD) environments. These data can support early disease detection and remote patient monitoring. However, wearable data are often noisy, sensitive, and distributed across different devices. This paper proposes a multimodal neuro-symbolic model to overcome these limitations and incorporates it into a secure Edge–Fog–Cloud framework for anomaly detection in smart healthcare applications. The proposed system integrates the semantic analysis of clinical text using Bio-ClinicalBERT with temporal numerical data using an LSTM-based model, creating a unified neuro-symbolic artificial intelligence (AI) pipeline. Initial data processing is performed at the Edge, whereas inference is carried out at distributed Fog nodes for low-latency anomaly detection. Model training is handled in the Cloud, and privacy-preserving federated learning (FL) is supported through Homomorphic Encryption (HomEnc) to facilitate collaborative model training without sharing raw patient data. A sharded Tangle ledger is also used, with transactions broadcast by the Fog nodes and validated in the Cloud to create tamper-evident transaction logs. Furthermore, Honey Encryption (HoneyEnc) is integrated into the Fog layer to enhance security against brute-force attacks. Experimental results show that the proposed framework achieved 99.22% accuracy and a 99.31% F1-score on the held-out test set, with bootstrap 95% confidence intervals of 98.96–99.47% for accuracy and 99.08–99.53% for the F1-score. It also reduced detection latency from 185 ms in the baseline setting to approximately 50 ms in the Fog-inference setting. The blockchain layer achieved approximately 500 Transactions Per Second (TPS), while higher throughput was observed under increased transaction load and shard parallelism. Because the evaluation is based on synthetic multimodal EHR-like data and controlled simulations, the reported findings should be interpreted as proof-of-concept internal validation rather than evidence of deployment-ready clinical generalizability; external validation using real wearable biosensor data, hospital IoMT streams, or public clinical datasets such as MIMIC-III/MIMIC-IV is required before clinical deployment. These results highlight the potential of the proposed system for secure data processing and trustworthy anomaly detection in smart healthcare environments.

## 1. Introduction

The volume of healthcare data is expanding rapidly with the growing adoption of electronic health records (EHRs), wearable sensors, and a wide range of Internet of Medical Things (IoMT) devices operating in Internet of Disease (IoD) environments [1,2]. As healthcare data grow in scale and complexity, with multimodal systems becoming increasingly common, there are important opportunities to use real-time analytics and predictive models to improve patient care. More specifically, smart health applications, such as remote monitoring platforms and wearable diagnostic systems, can support the early and accurate detection of anomalies and enable timely interventions, leading to better outcomes [3,4]. One of the key issues for researchers is how to manage these multimodal data safely, efficiently, and in a privacy-preserving manner in resource-constrained environments.

Many existing anomaly detection systems do not fully satisfy the security requirements of smart healthcare systems [5,6,7]. Traditional statistical methods and conventional Machine Learning (ML) models may be unable to fully capture the complex temporal dynamics of physiological data or the semantic depth of clinical text [8,9]. Centralized cloud-based systems offer substantial computational power but may introduce high latency and privacy risks because raw data often need to be transmitted to cloud servers [10,11,12]. In contrast, edge-only or fully decentralized solutions may lack the computational capacity required for advanced multimodal inference or collaborative learning across diverse contexts, which is often supported by centralized processing hubs [13,14,15]. In addition, the integrity, transparency, and reliability of anomaly alerts and system updates remain unresolved issues in smart healthcare applications, where dependability and interpretability are of paramount importance [16,17]. These limitations indicate a need for an integrated smart healthcare framework that can simultaneously support multimodal anomaly detection, privacy-preserving collaborative learning, low-latency Fog-level inference, and tamper-evident auditability. Existing approaches generally address these requirements separately, leaving a gap for a unified architecture that combines intelligent health monitoring with secure and scalable distributed processing.

**Figure 1:**
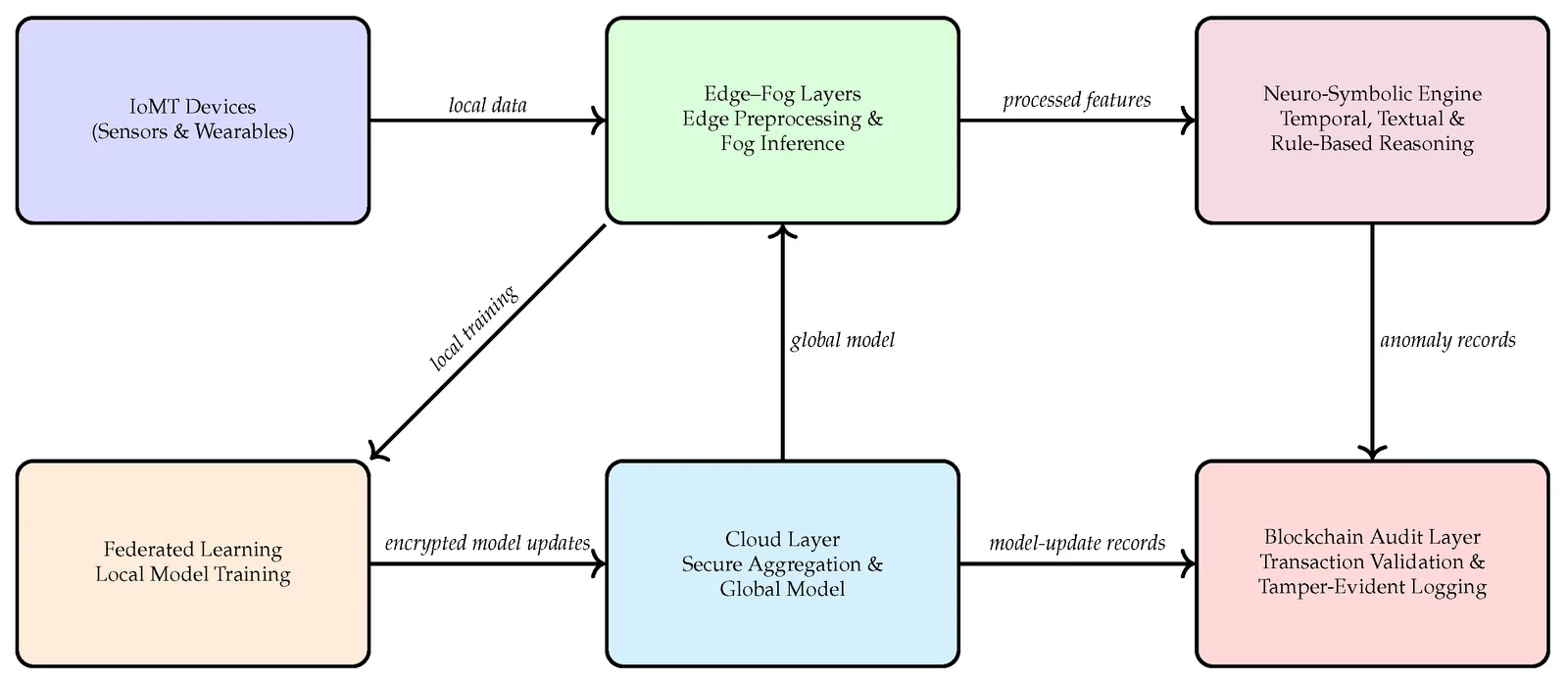
High-level conceptual overview of the proposed blockchain-enabled federated neuro-symbolic framework.

Numerous wearable biosensors are currently available for continuous health monitoring. They can measure heart rate, blood pressure, body temperature, glucose level, respiration rate, and oxygen saturation. These devices generate large volumes of data. These data must be processed quickly to detect abnormal health conditions. However, latency and privacy issues may arise when raw data are uploaded to the Cloud. Hence, a secure Edge–Fog–Cloud framework is required for reliable wearable health monitoring. To address these limitations, we propose an integrated framework for low-latency anomaly detection in smart healthcare settings. The proposed solution consists of a neuro-symbolic architecture that combines Long Short-Term Memory (LSTM) networks for temporal signal analysis with Bio-ClinicalBERT for contextual clinical-text representation. This architecture is integrated with a secure, multi-layer Edge–Fog–Cloud computing structure. Figure 1 presents a high-level conceptual overview of the proposed approach. The framework supports feature preprocessing at the Edge, low-latency inference at Fog nodes, and global model aggregation coordinated in the Cloud. To preserve patient privacy during collaborative training, federated learning (FL) is integrated with Homomorphic Encryption (HomEnc), allowing raw patient data to remain local. Additionally, a sharded Tangle ledger is used to record anomaly events and model-update records in a permissioned and tamper-evident audit trail, with transaction creation handled at the Fog layer and validation performed in the Cloud. This paper also explores the integration of Honey Encryption (HoneyEnc) to provide additional conceptual protection against brute-force guessing attacks. This work has the following main contributions:

1. A multimodal neuro-symbolic anomaly detection model is proposed by integrating the temporal analysis capabilities of an LSTM network with the clinical-text understanding capabilities of Bio-ClinicalBERT and clinically inspired symbolic consistency rules. The model combines physiological sensor information with clinical text to improve anomaly detection performance.
2. A privacy-preserving global training strategy integrates FL with HomEnc to support collaborative model training without sharing raw patient data. The layered Edge–Fog–Cloud architecture is designed to support low-latency and resource-aware processing in wearable biosensor-based smart healthcare applications.
3. A sharded Tangle ledger is integrated to support decentralized and tamper-evident logging of anomaly events and model-update records. The blockchain evaluation reports approximately 500 Transactions Per Second (TPS) and higher throughput under increased transaction load and shard parallelism.
4. The framework is designed to support continuous monitoring of physiological parameters obtained from medical and wearable sensors. HoneyEnc is incorporated into the Fog layer to provide conceptual protection against brute-force guessing attacks.
5. The framework is evaluated through controlled simulations to assess detection performance, latency, blockchain throughput, robustness, and privacy-related overheads, while external real-world clinical validation is identified as future work.
6. Further experiments and analyses include comparisons of several FL strategies, trained ablation studies examining the main learning components, quantitative explainability analysis using LIME stability and attention/symbolic-rule alignment, and blockchain scalability analysis.

The remainder of this paper is organized as follows. Section 2 reviews the related work, Section 3 presents the proposed architecture, Section 4 describes the methodology, Section 5 reports the experimental results, and Section 6 presents the conclusion and future work.

## 2. Related Work

X. Wang et al. [1] proposed a hybrid AI-blockchain solution for the Internet hospital setting, combining improved RSA for secure data transmission, Isolation Forest for real-time anomaly detection, and a private blockchain system based on Proof-of-Authority. However, the approach only supports numerical IoT streams and relies on a cloud-based execution model, which does not support multimodal data fusion or Fog-layer inference. S. Khan et al. [18] introduced a blockchain-supported FL system that allows hospitals to train shared models using their EMRs while preserving the privacy of their data. Data ownership is maintained, access is secured through smart contracts, and model updates are recorded on the blockchain. The study, however, relies on offline EMR datasets and does not consider real-time IoT data or Edge-level latency requirements. X. Wang et al. [19] implemented an alliance blockchain with enhanced RSA, achieving 24.7% higher throughput and 19.8% lower latency for IoT sensor data in Internet hospitals. However, the private-chain setup carries the risk of concentrating control and reducing transparency as data volumes increase.

V. Salunkhe et al. [20] integrated blockchain with Filecoin-powered IPFS and zero-knowledge proofs to secure and scale EMR management. Their design allows patients to choose between light- and full-node roles but is validated only through system-level benchmarks, leaving real-world latency unexplored. R. Vijay Anand et al. [21] designed a PoA-based blockchain framework that combines FL, LSTM autoencoders, and smart-contract-based model management. D. Dhinakaran et al. [22] proposed the Adaptive Feature-Centric Polynomial Data-Security Model (AFPDSM), which encrypts EHRs according to data-importance categories and reports 98% security gains, although encryption and decryption still require approximately 100 s per batch and incur considerable computational overhead. M. Alazab [23] introduced an adaptive hypervisor-security protocol that combines federated learning-based anomaly detection with cloud-security monitoring. The study, however, is evaluated only in a simulated cloud setting and focuses on VM-level telemetry, leaving real-world deployment and heterogeneous-workload validation for future work.

V. Karthikeyan et al. [24] reviewed three blockchain-based healthcare solutions featuring identity-based access, smart-contract-based exchange, and immutable audit trails. They highlighted both the security benefits of these solutions and the remaining challenges related to scalability and interoperability. In a related direction, S. B. Sangeetha et al. [25] presented the Secure Healthcare Access Control System (SHACS), which incorporates role-, attribute-, and rule-based policies together with real-time anomaly detection on the MIMIC-III dataset, reducing authentication time and access delay by 30% and 25%, respectively, compared with traditional methods. However, SHACS only addresses centrally managed access to EHRs and does not integrate blockchain or distributed enforcement at the Fog level. J. Wang et al. [26] proposed a deep-learning approach for detecting abnormal traffic in IoMT-blockchain networks using grouped feature extraction, multiple autoencoders, and residual learning to identify temporal patterns. However, the study does not consider privacy-preserving training or real-time Edge-deployment requirements. Recent fault-diagnosis research also highlights challenges relevant to robust intelligent health-monitoring systems. Gao et al. [27] proposed a domain feature decoupling network (DFDN) that separates domain-related and fault-related representations to improve generalization under unseen operating conditions. Li et al. [28] developed a joint collaborative adaptation network (JCAN) that combines multi-source domain adaptation, class-aware learning, and collaborative decision fusion to address class imbalance and variable operating conditions. Although these methods target rotating machinery and bearing fault diagnosis rather than healthcare monitoring, they demonstrate the importance of domain generalization, robustness to distribution shifts, and class-aware learning when intelligent diagnostic models are applied under heterogeneous and changing operating conditions.

Despite these advances, three main gaps persist: most prior frameworks either (i) operate on single-modal or purely numerical data streams, (ii) confine inference and consensus to Cloud or consortium layers, or (iii) lack integrated privacy-preserving learning and high-throughput ledger support (see Table 1). Few studies simultaneously address multimodal fusion, low-latency Fog-level inference, federated aggregation with HomEnc, and sharded-ledger scalability. These gaps motivate our proposed Edge–Fog–Cloud architecture, which unifies neuro-symbolic anomaly detection, privacy-preserving FL, and a sharded Tangle ledger to support low-latency and trustworthy analytics in smart healthcare applications under controlled simulation settings.

## 3. Threat Model and Proposed Architecture

### 3.1. Threat Model

Let a typical smart healthcare network be represented as a heterogeneous cyber–physical system:

(1) G=(D,H,S,P,R),

where $D=\{D_{1},D_{2},\ldots ,D_{N}\}$ denotes the set of patient-side IoMT devices, $H=\{H_{1},H_{2},\ldots ,H_{M}\}$ denotes the set of hospital-network and clinical-infrastructure components, $S=\{S_{1},S_{2},\ldots ,S_{K}\}$ denotes the set of external or cloud-based healthcare services, P represents the set of patients and clinical users, and R represents the set of healthcare records, logs, and audit trails. The IoMT device set D may include wearable sensors, smartwatches, bedside monitors, infusion pumps, smart hospital beds, and clinician tablets. The hospital infrastructure set H may include wireless access points, hospital gateways, nurse-station servers, EHR/database servers, and monitoring workstations. The external service set S may include cloud servers, remote physician access systems, audit-log databases, and telemedicine or mobile-application services.

At time *t*, a patient-side device $D_{i}$ generates a healthcare observation:

(2) $$x_{i}^{t}=\left(v_{i}^{t},m_{i}^{t},l_{i}^{t}\right),$$

where $v_{i}^{t}$ denotes physiological or vital-sign data, $m_{i}^{t}$ denotes device metadata, and $l_{i}^{t}$ denotes local event or operational logs. These observations are transmitted through the hospital network to clinical-infrastructure components and, when required, to external or cloud-based healthcare services. A typical communication path is represented as

(3) $$D_{i}\to H_{j}\to S_{k},$$

where $D_{i}\in D$, $H_{j}\in H$, and $S_{k}\in S$. This communication may support monitoring, diagnosis, EHR updates, remote consultations, audit logging, and telemedicine.

The adversary is denoted by A. We assume that A is a probabilistic polynomial-time adversary that can observe, intercept, replay, modify, or disrupt selected communication links in the smart healthcare network. The adversary may also compromise a subset of IoMT devices, hospital-infrastructure components, or external service interfaces.

Let $D_{c}\subseteq D$, $H_{c}\subseteq H$, and $S_{c}\subseteq S$ denote the compromised IoMT devices, hospital-infrastructure components, and external service interfaces, respectively. Their corresponding honest subsets are defined as

(4) $$D_{h}=D\setminus D_{c},\;H_{h}=H\setminus H_{c},\;S_{h}=S\setminus S_{c}.$$

The threat surface is distributed across devices, communication links, clinical servers, record repositories, and external access points. Let $L_{DH}$ denote the communication links between patient-side IoMT devices and hospital infrastructure, and let $L_{HS}$ denote the communication links between hospital infrastructure and external or cloud services. The total attack surface can be represented as

(5) $$\Omega =D\cup H\cup S\cup L_{DH}\cup L_{HS}\cup R.$$

An attack is an attempt by A against any element ω∈Ω to compromise the confidentiality, integrity, availability, or trustworthiness of a healthcare operation.

The first threat is data interception, in which the adversary attempts to obtain sensitive healthcare information transmitted between devices and hospital infrastructure:

(6) $$A:\left\{x_{i}^{t},L_{DH}\right\}\to {\hat{x}}_{i}^{t}.$$

In this case, ${\hat{x}}_{i}^{t}$ represents the adversary’s estimate of the patient information. When the estimation accuracy is sufficiently high, patient privacy may be compromised.

The second threat is replay or modification, in which the adversary receives a legitimate message $x_{i}^{t}$ and either replays it at a later time or modifies it before delivery:

(7) $$x_{i}^{t}\to {\tilde{x}}_{i}^{t^{\prime }},$$

where ${\tilde{x}}_{i}^{t^{\prime }}$ represents a replayed or modified observation at time $t^{\prime }$. This manipulation may mislead monitoring systems and result in an incorrect interpretation of the clinical situation.

The third threat is device compromise. Once the adversary has compromised an IoMT device $D_{i}$, it can modify the behavior of the device, corrupt its output, or exploit it to gain access to the hospital network:

(8) $$D_{i}\in D_{c}\Rightarrow x_{i}^{t}\neq x_{i,\mathrm{true}}^{t},$$

where $x_{i,\mathrm{true}}^{t}$ represents the true physiological measurement and $x_{i}^{t}$ represents the value transmitted by the compromised device.

Unauthorized access is the fourth threat, in which the adversary attempts to gain unauthorized access to clinical infrastructure or external services:

(9) $$\mathrm{Access}\left(A,H_{j}\right)=1\;\mathrm{or}\;\mathrm{Access}\left(A,S_{k}\right)=1,$$

where $H_{j}\in H$ and $S_{k}\in S$. An access breach may compromise patient records, monitoring dashboards, clinical workstations, or remote-consultation services.

The fifth threat is false data or alert insertion. In this scenario, the adversary injects fabricated clinical readings or spoofed alert messages into the monitoring flow:

(10) $$A:⌀\to x_{\mathrm{fake}}^{t},$$

where $x_{\mathrm{fake}}^{t}$ denotes a fake observation or alert. These attacks may trigger false alarms, conceal genuine anomalies, force unnecessary interventions, or delay clinical responses.

Record tampering is the sixth threat. The adversary may attempt to alter EHR data, audit trails, or monitoring records:

(11) $$r_{j}\to r_{j}^{\prime },$$

where $r_{j}\in R$ is an original healthcare record and $r_{j}^{\prime }$ is its modified version. The integrity violation is detected if

(12) $$H\left(r_{j}\right)\neq H\left(r_{j}^{\prime }\right),$$

where H(·) denotes a cryptographic hash function.

The seventh threat is denial-of-service or latency disruption. The adversary may disrupt communication paths, hospital gateways, EHR/database servers, or external services, causing delays in healthcare operations. The total network latency is represented as

(13) $$T_{\mathrm{net}}=T_{DH}+T_{H}+T_{HS},$$

where $T_{DH}$ is the communication delay between IoMT devices and hospital infrastructure, $T_{H}$ is the processing delay in the hospital network, and $T_{HS}$ is the delay between hospital infrastructure and external services. A latency attack is considered successful if

(14) $$T_{\mathrm{net}}>T_{\max},$$

where $T_{\max}$ is the maximum acceptable delay for clinical monitoring or response.

The eighth threat is privacy leakage, in which the adversary attempts to infer sensitive patient attributes from intercepted data, device behavior, access patterns, or stored records:

(15) $$A:\left\{x_{i}^{t},m_{i}^{t},l_{i}^{t},r_{j}\right\}\to p_{i},$$

where $p_{i}$ represents private patient information. The privacy objective is to ensure that

(16) $$\mathrm{Pr}\;\left[A\left(x_{i}^{t},m_{i}^{t},l_{i}^{t},r_{j}\right)=p_{i}\right]\leq \epsilon ,$$

where ϵ is a small probability.

**Figure 2:**
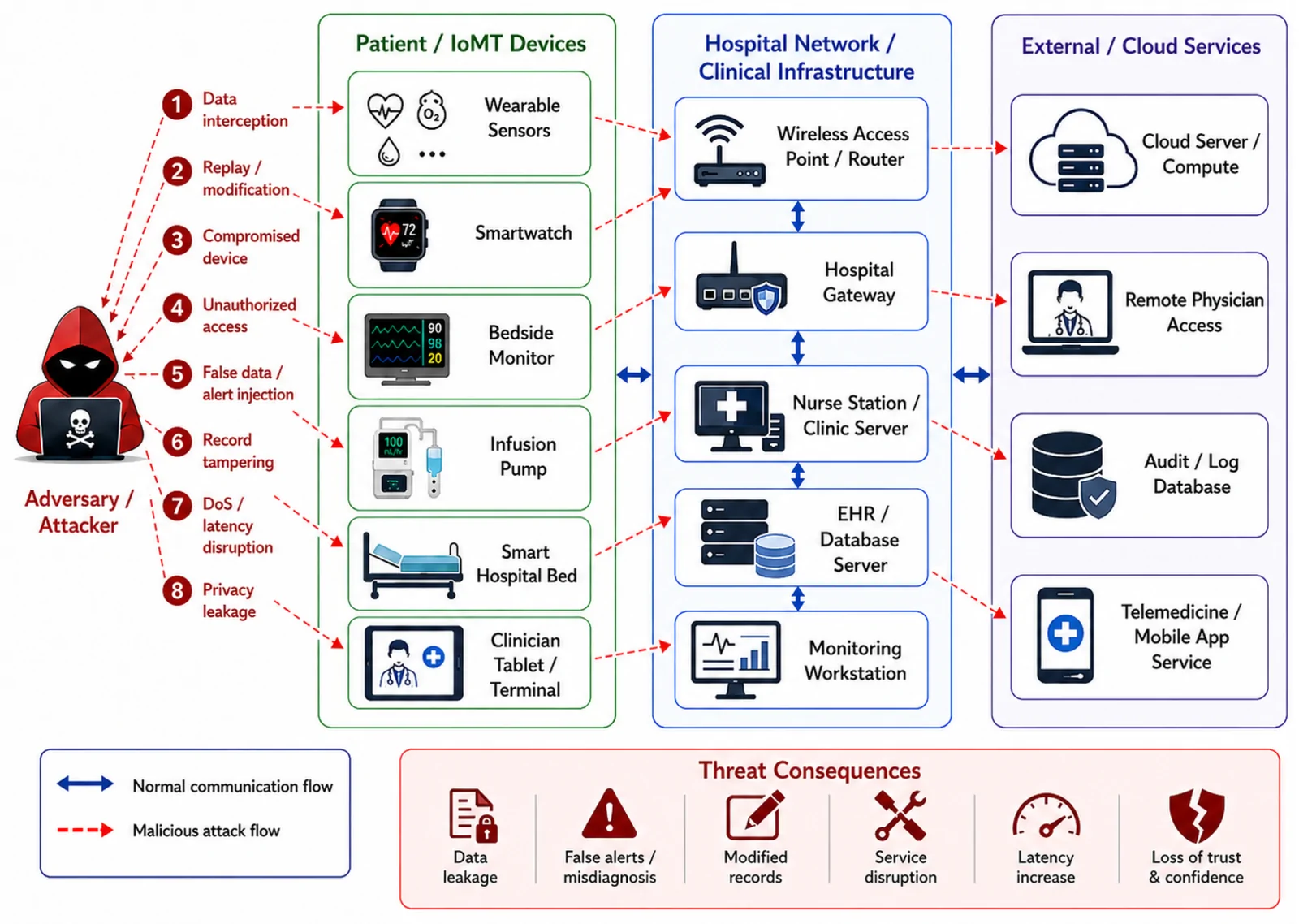
Threat model for a typical smart healthcare network.

The security objectives of the smart healthcare threat model are formally defined as

(17) $$S_{\mathrm{obj}}=\left\{S_{\mathrm{conf}},S_{\mathrm{int}},S_{\mathrm{auth}},S_{\mathrm{avail}},S_{\mathrm{trust}}\right\}.$$

where $S_{\mathrm{conf}}$ represents the confidentiality of patient data, $S_{\mathrm{int}}$ represents the integrity of clinical readings and healthcare records, $S_{\mathrm{auth}}$ represents authorized access to clinical systems and remote services, $S_{\mathrm{avail}}$ represents the availability of monitoring and clinical operations, and $S_{\mathrm{trust}}$ represents the trustworthiness of alerts, logs, and decision-support outputs. These objectives correspond to the major threat consequences shown in Figure 2, including data leakage, false alerts or misdiagnosis, modified records, service disruption, increased latency, and loss of trust.

Therefore, the presented threat model characterizes a typical smart healthcare network as a distributed attack surface composed of IoMT devices, hospital infrastructure, external or cloud services, communication links, and healthcare records. The adversary may target any of these components to compromise confidentiality, integrity, availability, authentication, or trust. This formulation provides the basis for designing and assessing the proposed secure healthcare framework under the defined cyber–physical threat scenarios.

### 3.2. Architecture

**Figure 3:**
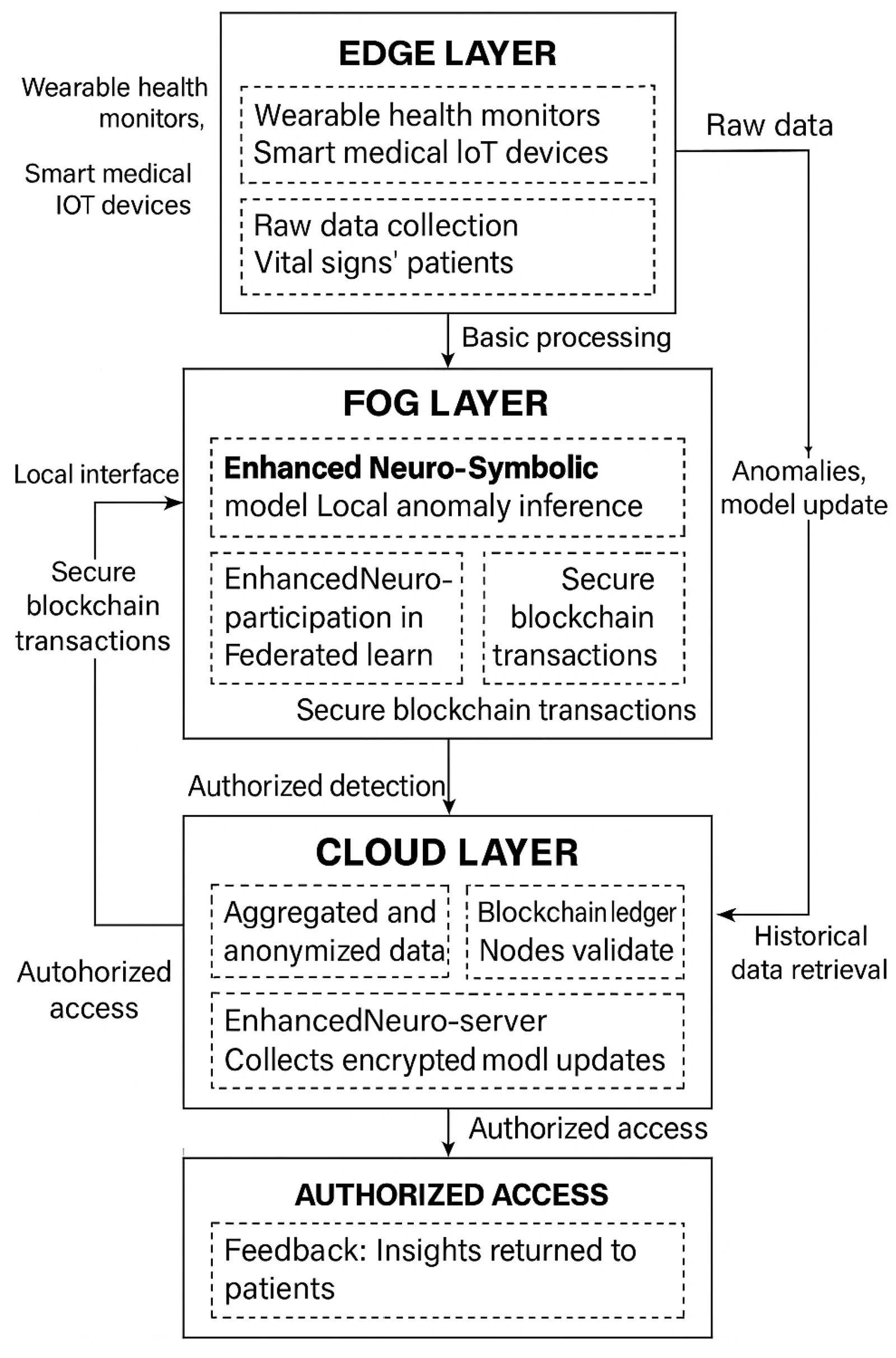
Blockchain-enabled federated neuro-symbolic anomaly-detection framework across the Edge–Fog–Cloud continuum.

To address the challenges of low-latency anomaly detection, data privacy, security, and scalability in smart healthcare environments, we propose a multilayered architecture that integrates Edge, Fog, and Cloud computing. The architecture is further augmented with neuro-symbolic AI, FL, and blockchain technologies. It is designed to optimize data processing and model training by distributing tasks according to their proximity to the data source, computational requirements, and security considerations. The proposed architecture, illustrated in Figure 3, includes three layers: the Edge layer, the Fog layer, and the Cloud layer. Each layer performs specific functions to ensure an effective and secure flow of healthcare data and anomaly-detection results.

The architecture facilitates efficient end-to-end data flow and model refinement. Raw data are collected at the Edge and sent to the Fog layer for preprocessing and low-latency anomaly detection. Only selected outputs are transmitted to the Cloud, including anomaly alerts, model updates, and transaction logs, thereby reducing relative bandwidth demand and limiting the exposure of raw data. FL operates in iterative training rounds, during which the global model is distributed from the Cloud to the Fog nodes. Each Fog node performs local training and sends encrypted model updates to the Cloud coordinator for secure aggregation. The Fog layer generates transactions, while the Cloud verifies them using a permissioned consortium blockchain in which validation is restricted to trusted healthcare actors, such as hospitals and cloud-based validators. This controlled environment supports data privacy and restricted access. A sharded Tangle-based directed acyclic graph (DAG) ledger is used to evaluate transaction throughput and latency under controlled simulation settings. Transactions generated by the Fog nodes are verified by trusted Cloud nodes and added as tamper-evident records. This allows the blockchain to act as a secure audit layer for anomaly events and federated-model updates, enabling activity to be tracked and verified without storing raw patient data on the ledger.

To clarify the interaction between FL and the blockchain layer, the Fog layer includes a lightweight blockchain gateway composed of four modules: a transaction builder, a signature module, a shard router, and a ledger verifier. After each local FL update or anomaly decision, the transaction builder serializes only verification metadata, such as the anomaly-event identifier, timestamp, local model-update hash, and Fog-node identifier. The signature module signs this metadata to verify the source of the update or event. The shard router assigns the transaction to a Tangle shard using the sharding function described in Section 4.6. The ledger verifier checks received transaction confirmations, validates hash consistency, and verifies whether the signed metadata has been accepted by the assigned Tangle shard. Raw patient records and full model parameters are not stored on the ledger; only hashes, signatures, timestamps, and references are logged for auditability. Secure aggregation is performed by the FL coordinator using HomEnc, while the blockchain gateway provides hash and signature verification, shard routing, replay resistance, and tamper-evident audit logging.

1. Edge Layer: Edge-layer devices are located closest to the data source and include wearable health monitors, smart IoMT devices, infusion pumps, smart beds, and local interfaces. These devices interact directly with patients or healthcare providers. The main function of the Edge layer is to collect raw healthcare data, such as vital signs, patient-reported symptoms, and device logs. Lightweight processing is performed locally, including data filtering and formatting, while MQTT is used for lightweight sensor communication and HTTPS supports secure data transmission between system layers. This processing prepares the acquired data for transmission to the Fog layer while avoiding unnecessary communication.
2. Fog Layer: The Fog layer consists of distributed computing nodes located closer to patients than the Cloud, including hospital servers, regional clinic hubs, and dedicated gateways. It offers greater processing capacity than Edge devices and supports data aggregation and local analytics. The Fog layer is a central component of low-latency operations. It receives inputs from several Edge devices and performs multimodal preprocessing, including temporal sequencing of physiological data and feature extraction from clinical notes. It performs local anomaly inference using the proposed neuro-symbolic model, allowing timely decisions to be made without transferring raw data outside the local healthcare environment. Each Fog node also participates in FL by training a local model using locally available data. If an anomaly or critical event is detected, the Fog node creates a signed blockchain transaction to record the event.
3. Cloud Layer: The Cloud layer provides a unified infrastructure with substantial computational and storage capacity. It acts as the global coordinator for federated learning and ledger validation. Aggregated information and audit metadata are stored securely in this layer, while encrypted model updates are received from Fog nodes. The FL coordinator is hosted in the Cloud and uses HomEnc to perform secure aggregation of local updates before periodically updating the global model. Additionally, the Cloud maintains blockchain validator nodes that validate transactions, implement consensus, and maintain a tamper-evident distributed ledger using a sharded Tangle design. It also supports authorized access for healthcare providers, researchers, and auditors to query the blockchain, review anomaly trends, and interact with aggregated system data. Conceptually, the Cloud also coordinates the HoneyEnc schemes deployed at the Fog layer.

## 4. Methodology

**Figure 4:**
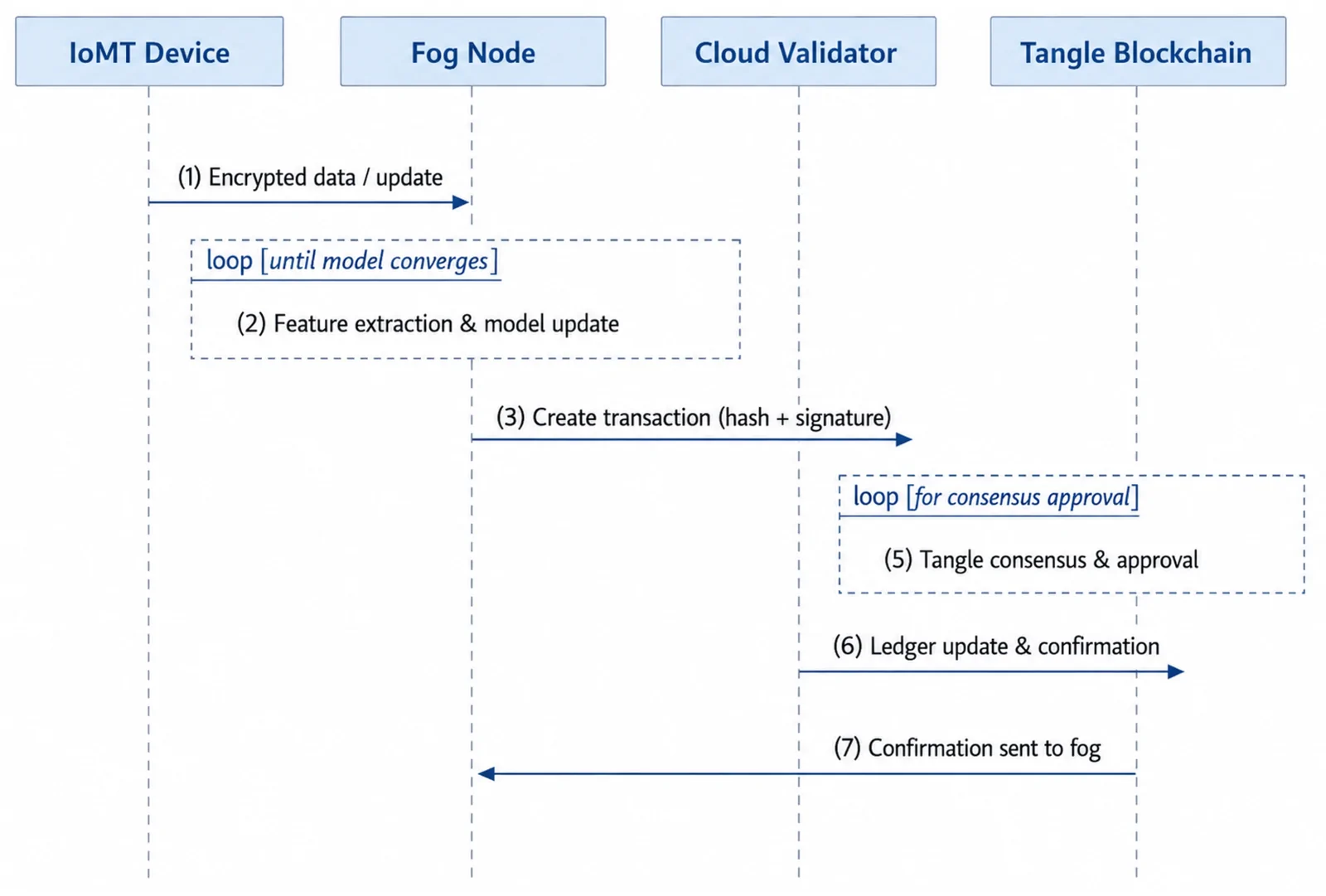
Sequential transaction flow in the proposed federated neuro-symbolic anomaly-detection framework.

This section presents the proposed methodology for low-latency anomaly detection in smart healthcare settings. The proposed pipeline jointly models (i) temporal physiological dynamics, (ii) contextual clinical semantics, and (iii) rule-consistent decision-making while supporting privacy-preserving collaborative training and tamper-evident auditability of security-critical events. The individual building blocks, including LSTM, Bio-ClinicalBERT, attention-based fusion, federated learning, Homomorphic Encryption, the sharded Tangle ledger, and Honey Encryption, are established techniques and are not claimed as individually novel contributions. The contribution of this work lies in their coordinated integration in a unified Edge–Fog–Cloud architecture that couples multimodal neuro-symbolic anomaly detection with privacy-preserving federated optimization, Fog-level inference, and tamper-evident blockchain-based auditability for wearable health-monitoring workflows. The methodology is organized into six stages: dataset construction, multimodal preprocessing, neuro-symbolic anomaly detection, encrypted federated optimization, sharded Tangle validation, and encryption-based privacy preservation. The sequential transaction-processing workflow is illustrated in Figure 4. The research design follows a controlled simulation-based proof-of-concept evaluation. The proposed framework is assessed through three linked stages: (i) multimodal anomaly-detection performance using a synthetic EHR-like dataset, (ii) privacy-preserving federated training with Fog clients and a separate Cloud coordinator, and (iii) system-level evaluation of Fog-inference latency, blockchain throughput, relative upstream bandwidth, robustness, and privacy-related overheads. This design supports component-level and end-to-end evaluation while keeping the reported findings explicitly in the controlled simulation setting.

### 4.1. Wearable Biosensor Data Acquisition

The proposed framework is designed to accept data from wearable biosensors and medical Internet of Things devices. These devices may include smartwatches, wearable electrocardiogram monitors, continuous glucose monitors (CGMs), pulse oximeters, and temperature monitors. Physiological data are continuously acquired by these sensors. Lightweight filtering and data formatting are performed at the Edge layer. The processed data are then transmitted to the Fog layer for multimodal preprocessing and low-latency anomaly detection. The evaluation in this study is performed using synthetic physiological data. The generated variables represent measurements commonly obtained from wearable and medical sensors. Future work will validate the framework using real wearable-sensor data and external clinical cohorts. Table 2 shows the relationship between the physiological features used in this study and their possible wearable-sensor sources.

### 4.2. Dataset Description

Clinical generalizability note. The present evaluation uses a synthetic multimodal EHR-like dataset to provide a controlled proof-of-concept assessment of the proposed framework. Although the generated records include temporal vital-sign patterns, aligned clinical-text entries, and clinically inspired anomaly thresholds, synthetic data cannot fully reproduce the heterogeneity of real hospital environments, including institution-specific documentation practices, device-calibration differences, missing-data mechanisms, patient comorbidities, and operational noise. Therefore, the reported results should be interpreted as simulation-based internal validation rather than evidence of deployment-ready clinical generalizability. External validation using heterogeneous real-world clinical cohorts, such as MIMIC-III/MIMIC-IV or hospital IoMT streams, is identified as a necessary next step.

Let the prepared dataset be

(18) $$D={\left\{\left(X_{i},r_{i},y_{i}\right)\right\}}_{i=1}^{N}.$$

where $X_{i}$ denotes the numerical vital-sign sequence, $r_{i}$ denotes the aligned clinical text, and $y_{i}\in 0,1$ is the class label, where 0 represents normal and 1 represents anomaly.

Each physiological record for patient *i* at time *t* is represented as

(19) $$x_{i}^{t}=\left[x_{i,\mathrm{hr}}^{t},x_{i,\mathrm{sys}}^{t},x_{i,\mathrm{dia}}^{t},x_{i,\mathrm{glu}}^{t},x_{i,\mathrm{temp}}^{t},x_{i,\mathrm{resp}}^{t},x_{i,{\mathrm{SpO}}_{2}}^{t}\right]\in R^{7}.$$

A sliding window of size T=5 produces

(20) $$I_{i}^{t}=\left(X_{i}^{t},r_{i}^{t+1},y_{i}^{t+1}\right).$$

Algorithm 1 formalizes the temporal windowing and cross-modal alignment procedure, ensuring that each vital-sign sequence is paired with the corresponding clinical text and label. The train/test split satisfies

(21) $$D_{\mathrm{train}}\cap D_{\mathrm{test}}=⌀,\;D_{\mathrm{train}}\cup D_{\mathrm{test}}=D.$$

**Algorithm 1** Sliding-Window Construction and Multimodal Alignment**Require:** Patient records ${\left\{\left(x_{i}^{t},r_{i}^{t},y_{i}^{t}\right)\right\}}_{t=1}^{T_{i}}$, window size *T* **Ensure:** Aligned instances $\left\{I_{i}^{t}\right\}$1:**for** $t=T,\ldots ,T_{i}-1$ **do**2:      $X_{i}^{t}\leftarrow \left[x_{i}^{t-T+1},\ldots ,x_{i}^{t}\right]$3:      $I_{i}^{t}\leftarrow \left(X_{i}^{t},r_{i}^{t+1},y_{i}^{t+1}\right)$4:**end for**

Note: Table 3 reports the class distribution of the raw training records before sliding-window construction, whereas Table 4 reports the prepared sequential samples after windowing.

### 4.3. Multimodal Preprocessing

Multimodal preprocessing ensures temporal alignment between physiological windows and their corresponding clinical text. This alignment is important because clinical notes may be recorded sparsely, whereas vital signs are recorded continuously. Therefore, the pipeline explicitly associates each temporal context window with the subsequent clinical entry and label.

For a batch size of *B*, the numerical input tensor is represented as

(22) $$X\in R^{B\times T\times F},$$

where T=5 and F=7. Each clinical text $r_{i}$ is tokenized as

(23) $$q_{i}=\mathrm{Tok}\left(r_{i}\right)=\left[q_{i,1},q_{i,2},\ldots ,q_{i,L}\right].$$

where L=256 after truncation or padding. The resulting multimodal input is

(24) $$M_{i}=\left(X_{i},q_{i}\right).$$

### 4.4. Multimodal Neuro-Symbolic Anomaly Detection Model

Purely statistical thresholds may fail to capture complex temporal patterns, whereas purely deep-learning models may produce clinically inconsistent alerts that are difficult to interpret. In the considered setting, both predictive accuracy and clinical consistency are important. Therefore, we integrate a data-driven multimodal predictor with a neuro-symbolic constraint layer. The proposed detector combines temporal LSTM encoding, biomedical text representations obtained using Bio-ClinicalBERT, attention-based text aggregation, and a symbolic consistency penalty based on clinically inspired constraints during model optimization. The anomaly detector is represented as

(25) $$f_{\Theta }:R^{T\times F}\times N^{L}\to {\left[0,1\right]}^{2},$$

where Θ denotes the parameters of the temporal encoder, text encoder, attention module, fusion layer, and classifier. The two output values represent the predicted probabilities of the normal and anomaly classes and sum to one.

#### 4.4.1. Temporal Encoder (LSTM)

The LSTM models temporal dependencies in vital-sign streams, including gradual changes, transient variations, and delayed physiological responses. Given

(26) $$X_{i}=\left[x_{i}^{1},\ldots ,x_{i}^{T}\right],$$

the LSTM recurrence is defined as

(27) $$h_{i,t}^{L}=\mathrm{LSTM}\left(x_{i}^{t},h_{i,t-1}^{L};\Theta_{L}\right),\;t=1,\ldots ,T.$$

The temporal embedding is obtained from the final hidden state:

(28) $$z_{i}^{L}=h_{i,T}^{L}.$$

#### 4.4.2. Clinical Text Encoder (Bio-ClinicalBERT)

Clinical text, including descriptions of symptoms, medical conditions, and medications, may provide additional context for interpreting physiological measurements. Bio-ClinicalBERT produces contextual token representations as follows:

(29) $$H_{i}^{B}=\left[h_{i,1}^{B},h_{i,2}^{B},\ldots ,h_{i,L}^{B}\right]=\mathrm{BioClinicalBERT}\left(q_{i};\Theta_{B}\right).$$

#### 4.4.3. Attention-Based Text Representation

The attention mechanism assigns different weights to the token representations so that tokens that may be relevant to the prediction, such as ‘chest pain,’ ‘hypoglycemia,’ and ‘shortness of breath,’ can contribute differently to the aggregated text representation. The attention score and corresponding weight are calculated as

(30) $$e_{i,l}=v_{a}^{T}\tanh\left(W_{a}h_{i,l}^{B}+b_{a}\right),$$

and

(31) $$\alpha_{i,l}=\frac{\exp(e_{i,l})}{\sum_{m=1}^{L}\exp\left(e_{i,m}\right)}.$$

The pooled text embedding is then calculated as

(32) $$z_{i}^{B}=\sum_{l=1}^{L}\alpha_{i,l}h_{i,l}^{B}.$$

#### 4.4.4. Multimodal Fusion and Regularization

The fusion layer combines the temporal physiological representation $z_{i}^{L}$ with the clinical-text representation $z_{i}^{B}$. This allows the model to consider physiological measurements and their associated clinical context jointly. The representations are concatenated and projected as

(33) $$u_{i}=\left[z_{i}^{L};z_{i}^{B}\right],$$

and

(34) $$z_{i}^{F}=\mathrm{ReLU}\left(W_{F}u_{i}+b_{F}\right).$$

Dropout is applied during training to reduce overfitting:

(35) $${\tilde{z}}_{i}^{F}=\frac{m_{i}⊙z_{i}^{F}}{1-\rho },\;m_{i}∼\mathrm{Bernoulli}\left(1-\rho \right),$$

where ρ is the dropout rate and ⊙ denotes element-wise multiplication. During inference, dropout is disabled and ${\tilde{z}}_{i}^{F}=z_{i}^{F}$.

#### 4.4.5. Neuro-Symbolic Constraint Layer

A set of interpretable neuro-symbolic constraints is included to improve the consistency of anomaly predictions with clinically inspired vital-sign thresholds. The rules are derived from commonly used abnormal vital-sign ranges. They are not presented as independently clinician-validated diagnostic rules; formal clinician-in-the-loop validation is identified as future work.

Let the rule set be

(36) $$R=\left\{R_{1},R_{2},\ldots ,R_{K}\right\}.$$

where K=8 in the current implementation. Each rule operates on the final time step of the physiological input window X∗i, denoted by x∗i,T. The complete rule set is presented in Table 5.

These threshold rules provide interpretable abnormality cues and are not intended to replace clinical judgment. Their purpose is to regularize the neural model toward physiologically meaningful patterns. For real clinical use, the thresholds would require review and refinement by clinicians according to the patient population, clinical specialty, device calibration, and institutional practice.

Each rule $R_{k}$ is represented as a tuple

(37) $$R_{k}=\left(j_{k},s_{k},\tau_{k}\right).$$

where $j_{k}$ denotes the feature index, $s_{k}\in +1,-1$ indicates the direction of abnormality, and $\tau_{k}$ denotes the corresponding threshold. Upper-bound rules use $s_{k}=+1$, whereas lower-bound rules use $s_{k}=-1$.

Each rule is converted into a differentiable abnormality-activation score:

(38) $$g_{i,k}=\sigma \left(\beta s_{k}\left(x_{i,T,j_{k}}-\tau_{k}\right)\right).$$

where σ(·) is the sigmoid function and β is a smoothness parameter. In the current implementation, β=1. A value of $g_{i,k}$ close to 1 indicates strong activation of the corresponding abnormality condition, whereas a value close to 0 indicates weak or absent activation.

The individual rule activations are combined using a differentiable soft-OR operation:

(39) $$r_{i}=1-\prod_{k=1}^{K}\left(1-g_{i,k}\right).$$

where $r_{i}\in \left[0,1\right]$ represents the aggregated abnormality evidence produced by the rule set. This formulation does not require every rule to be active; a high value of any relevant rule can increase the aggregated abnormality evidence.

The symbolic consistency loss aligns the predicted anomaly probability $p_{i,1}$ with the aggregated rule evidence:

(40) $$L_{\mathrm{sym}}=\frac{1}{B}\sum_{i=1}^{B}{\left(p_{i,1}-r_{i}\right)}^{2}.$$

Because $L_{\mathrm{sym}}$ depends on the predicted anomaly probability, it contributes gradients to the neural-model parameters during backpropagation. It therefore encourages consistency between the neural prediction and the clinically inspired abnormality evidence without forcing all rules to activate.

Algorithm 2 summarizes the forward computation of the multimodal neuro-symbolic detector. It combines temporal physiological information obtained using the LSTM with contextual clinical-text representations obtained using Bio-ClinicalBERT. Attention-based aggregation and nonlinear projection are then used to calculate the class probabilities. The rule activations provide the abnormality evidence used by the symbolic consistency loss during model training.
**Algorithm 2** Forward Computation of the Multimodal Neuro-Symbolic Detector**Require:** $X_{i}\in R^{T\times F}$, $q_{i}\in N^{L}$, model parameters Θ, rule set R **Ensure:** ${\hat{y}}_{i}$, $p_{i}$, $r_{i}$  1:$z_{i}^{L}\leftarrow \mathrm{LSTM}\left(X_{i};\Theta_{L}\right)$                           ▹ temporal embedding  2:$H_{i}^{B}\leftarrow \mathrm{BioClinicalBERT}\left(q_{i};\Theta_{B}\right)$                        ▹ token embeddings  3:**for** l=1,…,L **do**  4:      $e_{i,l}\leftarrow v_{a}^{T}\tanh\left(W_{a}h_{i,l}^{B}+b_{a}\right)$  5:**end for**  6:$\alpha_{i,l}\leftarrow \frac{\exp(e_{i,l})}{\sum_{m=1}^{L}\exp\left(e_{i,m}\right)}$,    l=1,…,L  7:$z_{i}^{B}\leftarrow \sum_{l=1}^{L}\alpha_{i,l}h_{i,l}^{B}$                          ▹ attention-based aggregation  8:$z_{i}^{F}\leftarrow \mathrm{ReLU}\left(W_{F}\left[z_{i}^{L};z_{i}^{B}\right]+b_{F}\right)$                         ▹ multimodal fusion  9:${\tilde{z}}_{i}^{F}\leftarrow \mathrm{Dropout}\left(z_{i}^{F};\rho \right)$10:$o_{i}\leftarrow W_{C}{\tilde{z}}_{i}^{F}+b_{C}$                                   ▹ logits11:$p_{i}\leftarrow \mathrm{softmax}\left(o_{i}\right)$                               ▹ class probabilities12:**for** k=1,…,K **do**13:      $g_{i,k}\leftarrow \sigma \left(\beta s_{k}\left(x_{i,T,j_{k}}-\tau_{k}\right)\right)$14:**end for**15:$r_{i}\leftarrow 1-\prod_{k=1}^{K}\left(1-g_{i,k}\right)$                       ▹ aggregated rule evidence16:${\hat{y}}_{i}\leftarrow \arg\max_{c\in \{0,1\}}p_{i,c}$                             ▹ predicted class

#### 4.4.6. Classifier and Decision Rule

Given the predicted class probabilities, the final class label is determined as follows:

(41) $${\hat{y}}_{i}=\arg\max_{c\in \{0,1\}}p_{i,c}.$$

#### 4.4.7. Optimization Objective

The model is optimized using a joint objective that combines focal loss, symbolic consistency loss, and $L_{2}$ regularization:

(42) $$L=L_{\mathrm{focal}}+\lambda_{\mathrm{sym}}L_{\mathrm{sym}}+\lambda_{2}{∥\Theta ∥}_{2}^{2},$$

where $\lambda_{\mathrm{sym}}$ controls the contribution of the symbolic consistency loss and $\lambda_{2}$ controls the $L_{2}$ regularization term.

### 4.5. Federated Optimization with HomEnc

In realistic healthcare environments, patient data cannot be centralized because of privacy regulations and institutional constraints. Therefore, the proposed framework uses federated optimization to support collaborative model improvement while keeping raw data local. In the controlled evaluation, federated training is across Fog clients and a Cloud coordinator, whereas encrypted aggregation is assessed separately using representative model-update vectors. Accordingly, the HomEnc formulation below describes the proposed encrypted aggregation mechanism and its component-level evaluation, rather than a full end-to-end encrypted FL deployment.

To protect update values during aggregation, the framework uses additive HomEnc, which supports aggregation in ciphertext space.

(43) $$\min_{w}L\left(w\right)=\sum_{k=1}^{K}\frac{n_{k}}{n}L_{k}\left(w\right),\;L_{k}\left(w\right)=\frac{1}{n_{k}}\sum_{i=1}^{n_{k}}\ell \left(f_{w}\left(M_{i}^{k}\right),y_{i}^{k}\right),$$

where $M_{i}^{k}$ denotes the *i*th multimodal sample held by client *k*, $n=\sum_{k=1}^{K}n_{k}$, and ℓ(·) is instantiated by $L_{\mathrm{NS}}$.

At round *r*, client *k* performs *E* local steps:

(44) $$w_{k}^{r,e+1}=w_{k}^{r,e}-\eta_{l}\nabla L_{k}\left(w_{k}^{r,e}\right),\;w_{k}^{r,0}=w^{r},$$

and sends the update $\Delta w_{k}^{r}=w_{k}^{r,E}-w^{r}$.

Encrypted updates and encrypted aggregation are defined as

(45) $$\begin{array}{cc} ⟦\Delta w_{k}^{r}⟧ & ={\mathrm{Enc}}_{pk}\left(\Delta w_{k}^{r}\right),\\ ⟦\Delta w^{r}⟧ & =\overset{K}{\underset{k=1}{⨁}}\left(\frac{n_{k}}{n}⊗⟦\Delta w_{k}^{r}⟧\right). \end{array}$$

Here, ⊕ denotes homomorphic addition between ciphertexts, whereas ⊗ denotes multiplication of an encrypted model update by the plain text aggregation weight $\frac{n_{k}}{n}$ in the encrypted domain.

The aggregated update is then decrypted and applied to the global model:

(46) $$\Delta w^{r}={\mathrm{Dec}}_{sk}\left(⟦\Delta w^{r}⟧\right),\;w^{r+1}=w^{r}+\eta_{g}\Delta w^{r}.$$

Algorithm 3 specifies the proposed privacy-preserving training workflow. In the experimental evaluation, raw records remain local to the clients, while the encrypted aggregation component is assessed separately using representative update vectors. The HomEnc results should therefore be interpreted as component-level validation of encrypted aggregation rather than evidence of a full end-to-end encrypted FL deployment.
**Algorithm 3** Proposed Encrypted Federated Optimization (HomEnc Aggregation)**Require:** Initial global model $w^{0}$, client sample counts ${\left\{n_{k}\right\}}_{k=1}^{K}$, clients {1,…,K}, rounds *R*, local steps *E*, learning rates $\eta_{l}$ and $\eta_{g}$, keys (pk,sk) **Ensure:** Updated global model $w^{R}$
  1:**for** r=0,…,R−1 **do**  2:      **for** k=1,…,K **do**  3:            $w_{k}^{r,0}\leftarrow w^{r}$  4:            **for** e=0,…,E−1 **do**  5:                 $w_{k}^{r,e+1}\leftarrow w_{k}^{r,e}-\eta_{l}\nabla L_{k}\left(w_{k}^{r,e}\right)$  6:            **end for**  7:            $\Delta w_{k}^{r}\leftarrow w_{k}^{r,E}-w^{r}$  8:            $⟦\Delta w_{k}^{r}⟧\leftarrow {\mathrm{Enc}}_{pk}\left(\Delta w_{k}^{r}\right)$  9:      **end for**10:      $⟦\Delta w^{r}⟧\leftarrow {⨁}_{k=1}^{K}\left(\frac{n_{k}}{n}⊗⟦\Delta w_{k}^{r}⟧\right)$11:      $\Delta w^{r}\leftarrow {\mathrm{Dec}}_{sk}\left(⟦\Delta w^{r}⟧\right)$12:      $w^{r+1}\leftarrow w^{r}+\eta_{g}\Delta w^{r}$13:**end for**


### 4.6. Sharded Tangle Ledger (Auditability and Integrity)

Tamper-evident logging is important for smart healthcare anomaly alerts and security-critical system updates because a malicious party may attempt to alter an alert, delete an incident record, or dispute a previously logged event. To address this risk, the proposed framework represents verification metadata as transactions in a sharded Tangle-based directed acyclic graph (DAG) audit layer. Fog nodes create transaction metadata, while trusted Cloud-side validators verify signatures and record transaction status. The ledger design is assessed through a controlled Python-based audit-log simulation using shards; it is not presented as a production deployment of a Tangle network. Each event generates a transaction:

(47) $$\tau_{j}=\left(ID_{j},t_{j},pid_{j},e_{j},H\left(D_{j}\right),F_{j},\sigma_{j}\right),$$

where $ID_{j}$ denotes the event identifier, $t_{j}$ denotes the timestamp, $pid_{j}$ denotes a pseudonymized patient or device identifier, $e_{j}$ denotes the event type, $H(D_{j})$ denotes the hash of the event data, $F_{j}$ denotes the Fog-node identifier, and $\sigma_{j}$ denotes the digital signature of the source node.

Sharding is characterized by

(48) $$\psi \left(\tau_{j}\right)=1+\left(H(pid_{j}∥e_{j})\;\mathrm{mod}\;Q\right),$$

where *Q* is the number of shards. In the proposed design, sharding is intended to distribute audit traffic across validator workloads rather than route all transaction checks through a single shard. This design is relevant when multiple Fog nodes generate anomaly alerts, event logs, and model-update records concurrently.

This comparison indicates that the proposed ledger should be interpreted as a distributed audit layer rather than a replacement for hospital EHR databases. The ledger stores only verification-oriented information, such as hashes, timestamps, signatures, anomaly-event identifiers, and model-update references. This design avoids placing raw clinical records on-chain while supporting post hoc auditability and tamper detection for security-critical events. In this regard a comparison of audit-log alternatives is presented in Table 6. However, to further clarify the relationship with mainstream blockchain families, the proposed sharded Tangle ledger is compared conceptually with commonly used blockchain deployment categories in Table 7. The comparison is qualitative because the present study evaluates a controlled Python-based audit-log simulation rather than deploying production Ethereum, Hyperledger Fabric, or IOTA nodes under identical network conditions.

As shown in Table 7, the proposed ledger is not intended to replace all mainstream blockchain solutions. Instead, it is designed for the specific case of high-frequency IoMT audit logging, where multiple Fog nodes may concurrently generate anomaly-event hashes, model-update references, timestamps, and signatures. In single-hospital deployments, an append-only secure database or a conventional permissioned blockchain may be sufficient. For multi-Fog or multi-institution scenarios, the proposed sharded Tangle design is intended to provide parallel audit logging and distributed verification without storing raw clinical records on-chain.

Algorithm 4 describes the conversion of security-critical healthcare events, such as anomaly alerts or system updates, into tamper-evident ledger records. Sharding partitions audit traffic across *Q* shards, while tip approval forms a DAG-based audit trail.
**Algorithm 4** Proposed Sharded Tangle Transaction Flow (Create-Shard-Approve-Append)**Require:** Event payload *D*, metadata M=(ID,t,pid,e,F), shard count *Q* **Ensure:** Transaction vertex $v_{j}$ recorded in the assigned Tangle shard
1:$\sigma \leftarrow {\mathrm{Sign}}_{sk}\left(ID,t,pid,e,H\left(D\right),F\right)$2:$\tau_{j}\leftarrow \left(ID,t,pid,e,H\left(D\right),F,\sigma \right)$                      ▹ create signed transaction3:q←1+(H(pid∥e)modQ)                  ▹ assign shard using the sharding key4:**if** $\mathrm{VerifySig}(\tau_{j})=0$ **then**5:     **reject** $\tau_{j}$; **return**                       ▹ invalid sender or modified transaction6:**end if**7:Select tips $\Pi \left(\tau_{j}\right)=\left\{\tau_{a},\tau_{b}\right\}\subseteq \mathrm{Tips}\left(T_{q}\right)$8:Add approval edges $\left(v_{j},v_{a}\right)$ and $\left(v_{j},v_{b}\right)$ to $E_{T}$                 ▹ DAG approval edges9:Append $v_{j}$ to $V_{T}$ and record the validation status of $\tau_{j}$


### 4.7. Encryption Mechanisms

To protect healthcare payloads during transmission, a plain text message *m* is encrypted as

(49) $$c={\mathrm{Enc}}_{k_{s}}\left(m\right),\;m={\mathrm{Dec}}_{k_{s}}\left(c\right),$$

where $k_{s}$ is a symmetric session key.

#### 4.7.1. Honey Encryption

HoneyEnc is included to increase uncertainty for brute-force attempts against sensitive healthcare data on constrained nodes. It is defined as

(50) $$c_{H}={\mathrm{HEnc}}_{k}\left(m\right),\;{\mathrm{HDec}}_{k}\left(c_{H}\right)=m,\;{\mathrm{HDec}}_{k^{\prime }}\left(c_{H}\right)=m_{d},\;k^{\prime }\neq k,$$

where $m_{d}$ is a plausible decoy message that increases adversarial uncertainty when an incorrect key is used.

#### 4.7.2. Key Management Protocol (HomEnc Keys)

To reduce insider risk at aggregation points, key generation and controlled decryption are organized through the proposed key-management service (KMS):

(51) $$\begin{array}{cc} \left(pk,sk\right) & \leftarrow \mathrm{KeyGen}(\lambda ),\\ C_{k} & \leftarrow {\mathrm{Enc}}_{pk}\left(\Delta w_{k}\right),\\ \mathrm{Agg} & \leftarrow \overset{K}{\underset{k=1}{⨁}}\left(\frac{n_{k}}{n}⊗C_{k}\right),\\ \Delta w_{\mathrm{global}} & \leftarrow {\mathrm{Dec}}_{sk}\left(\mathrm{Agg}\right), \end{array}$$

where λ is the security parameter, $n=\sum_{k=1}^{K}n_{k}$, and Agg denotes the aggregated ciphertext.

Algorithm 5 specifies the trust separation for HomEnc in the proposed workflow. By retaining the secret key sk in the KMS, the Cloud can aggregate ciphertexts without direct access to the decryption key, while controlled recovery supports global model updates from encrypted client contributions.
**Algorithm 5** Key-Management Workflow for HomEnc Aggregation**Require:** Security parameter λ, clients k∈{1,…,K}, client sample counts ${\left\{n_{k}\right\}}_{k=1}^{K}$ **Ensure:** Public-key distribution and controlled recovery of the aggregated update
  1:(pk,sk)←KeyGen(λ)                       ▹ generate HomEnc key pair  2:Distribute pk to all clients and retain sk in the KMS     ▹ secret key remains outside the Cloud  3:**for** k=1,…,K **do**  4:      Client *k* computes $\Delta w_{k}$ locally  5:      $C_{k}\leftarrow {\mathrm{Enc}}_{pk}\left(\Delta w_{k}\right)$  6:**end for**  7:$\mathrm{Agg}\leftarrow {⨁}_{k=1}^{K}\left(\frac{n_{k}}{n}⊗C_{k}\right)$                   ▹ ciphertext aggregation at the Cloud  8:$\Delta w_{\mathrm{global}}\leftarrow {\mathrm{Dec}}_{sk}\left(\mathrm{Agg}\right)$                     ▹ controlled plaintext recovery  9:Apply $\Delta w_{\mathrm{global}}$ to form the updated global model10:Distribute the updated global model to participating clients

## 5. Experimental Setup and Results

The complete framework was evaluated through a controlled simulation conducted in Python 3.10 on Google Colab Pro using an NVIDIA T4 GPU with 16 GB of memory. Data processing was performed using pandas 1.5 and NumPy 1.24. The deep-learning components were implemented using PyTorch 1.13 with CUDA 11.7 and Hugging Face Transformers 4.33. The emilyalsentzer/Bio_ClinicalBERT checkpoint was used as the clinical-text encoder. FL was simulated using Flower 1.7, while CKKS-based HomEnc operations were implemented using TenSEAL 0.3.17. The Fog clients were represented as separate workers in the controlled simulation environment, while a separate coordinator process performed encrypted global-model aggregation.

The blockchain evaluation was conducted using a Python-based sharded Tangle audit-log simulation. Worker processes represented the ledger shards, while transaction hashing and signing were implemented using the Python hashlib module and the cryptography 41.0 package. The transactions contained verification metadata, including event identifiers, timestamps, signatures, and model-update hashes. Blockchain throughput was evaluated under different shard configurations and transaction loads in the controlled simulation environment.

For reproducibility, the experimental procedure followed the methodological pipeline defined in Section 4. Physiological records were first converted into sequential samples using a sliding window of T=5 over seven vital-sign features, while the corresponding clinical text was tokenized to a maximum length of 256 tokens. The multimodal neuro-symbolic model was then trained using the hyperparameters summarized in Table 8, including an LSTM hidden size of 64, a dropout rate of 0.30, AdamW optimization with a learning rate of $1\times 10^{-4}$ and weight decay of $1\times 10^{-5}$, focal-loss parameters α=0.25 and γ=2.0, and a batch size of 128. Federated optimization was evaluated over 20 training rounds using Fog clients. The blockchain experiments evaluated different shard configurations and transaction loads under the controlled settings summarized in Table 9.

The software packages, model hyperparameters, and blockchain settings used in the experiments are summarized in Table 8.

### 5.1. Performance Metrics

**Figure 5:**
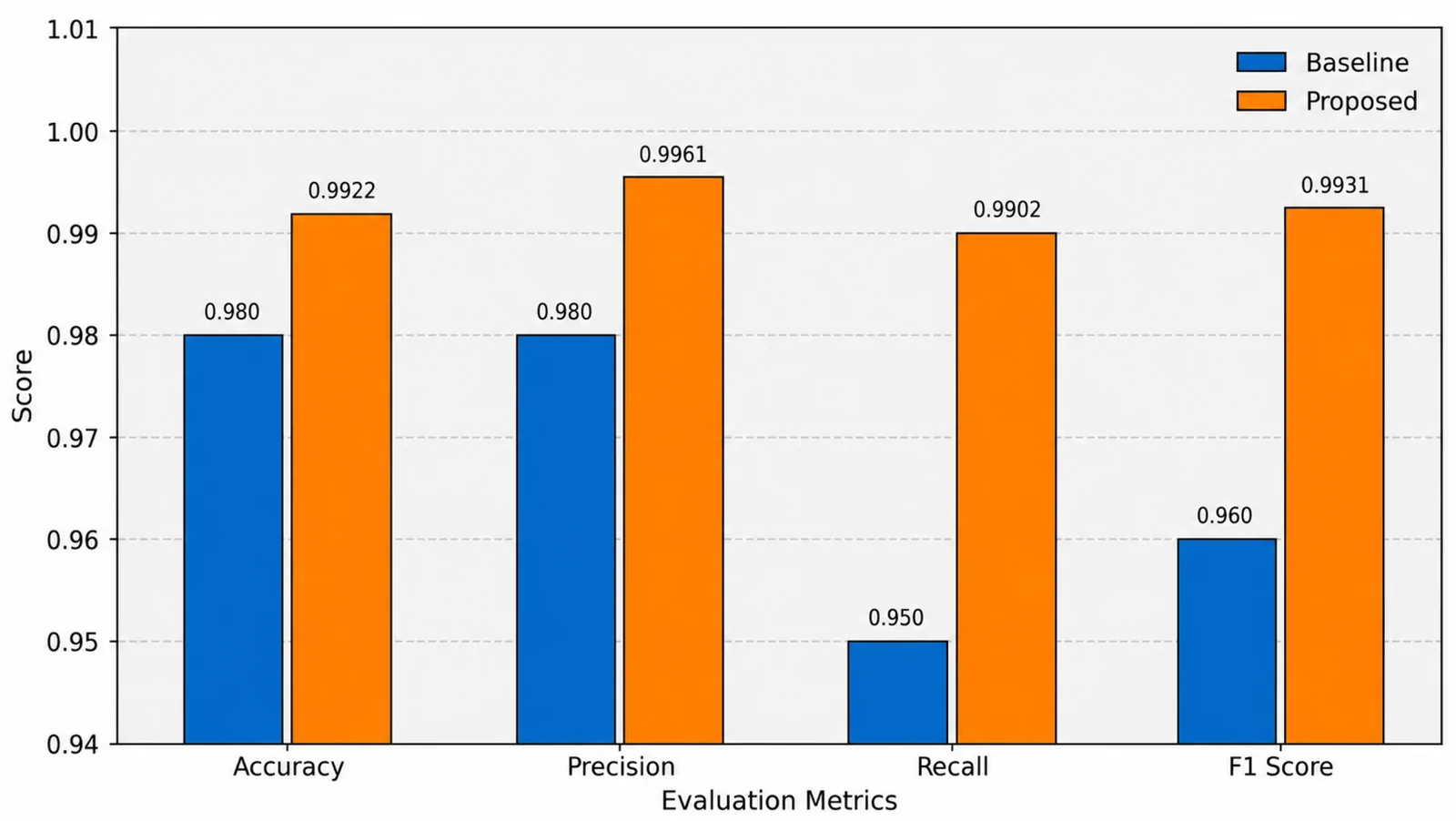
Performance comparison between the baseline anomaly detection model and the proposed blockchain-enabled federated neuro-symbolic framework.

The proposed multimodal framework achieved strong performance on the synthetic held-out test set, as shown in Figure 5. The overall accuracy was 0.9922. For the anomaly class, the model achieved a precision of 0.9961, a recall of 0.9902, and an F1-score of 0.9931. To complement these point estimates, we computed bootstrap 95% confidence intervals and performed five-fold patient-level cross-validation. These analyses assess the variability of the reported results under resampling and different data partitions. The results indicate high classification performance for the proposed multimodal model.

Compared with the baseline configuration shown in Figure 5, which achieved an accuracy of 0.9800, a precision of 0.9800, a recall of 0.9500, and an F1-score of 0.9600, the proposed model improved accuracy by 1.2 percentage points, precision by 1.6 percentage points, recall by 4.0 percentage points, and F1-score by 3.3 percentage points. The corresponding error rate decreased from 2.0% to approximately 0.8%, representing a relative error reduction of approximately 61%.

However, the high accuracy and F1-score should be interpreted cautiously because the synthetic anomaly labels were constructed using clinically inspired threshold-based patterns. This label construction may make the separation between normal and anomalous cases more explicit than in real-world clinical monitoring, where physiological variability, comorbidities, sensor noise, ambiguous clinical events, and uncertain labels can make anomaly detection substantially more difficult.

Table 10 presents the class-wise performance on the held-out test set. The model achieved a precision of 0.9873 and a recall of 0.9949 for normal records. For anomaly records, the precision and recall were 0.9961 and 0.9902, respectively. The macro-average and weighted-average scores were similar, indicating comparable performance across the two classes in this synthetic test set. Although the model is not perfectly accurate, the anomaly-class recall indicates that approximately 1 in 100 anomaly instances was missed in the held-out simulation.

### 5.2. Confusion Matrix

**Figure 6:**
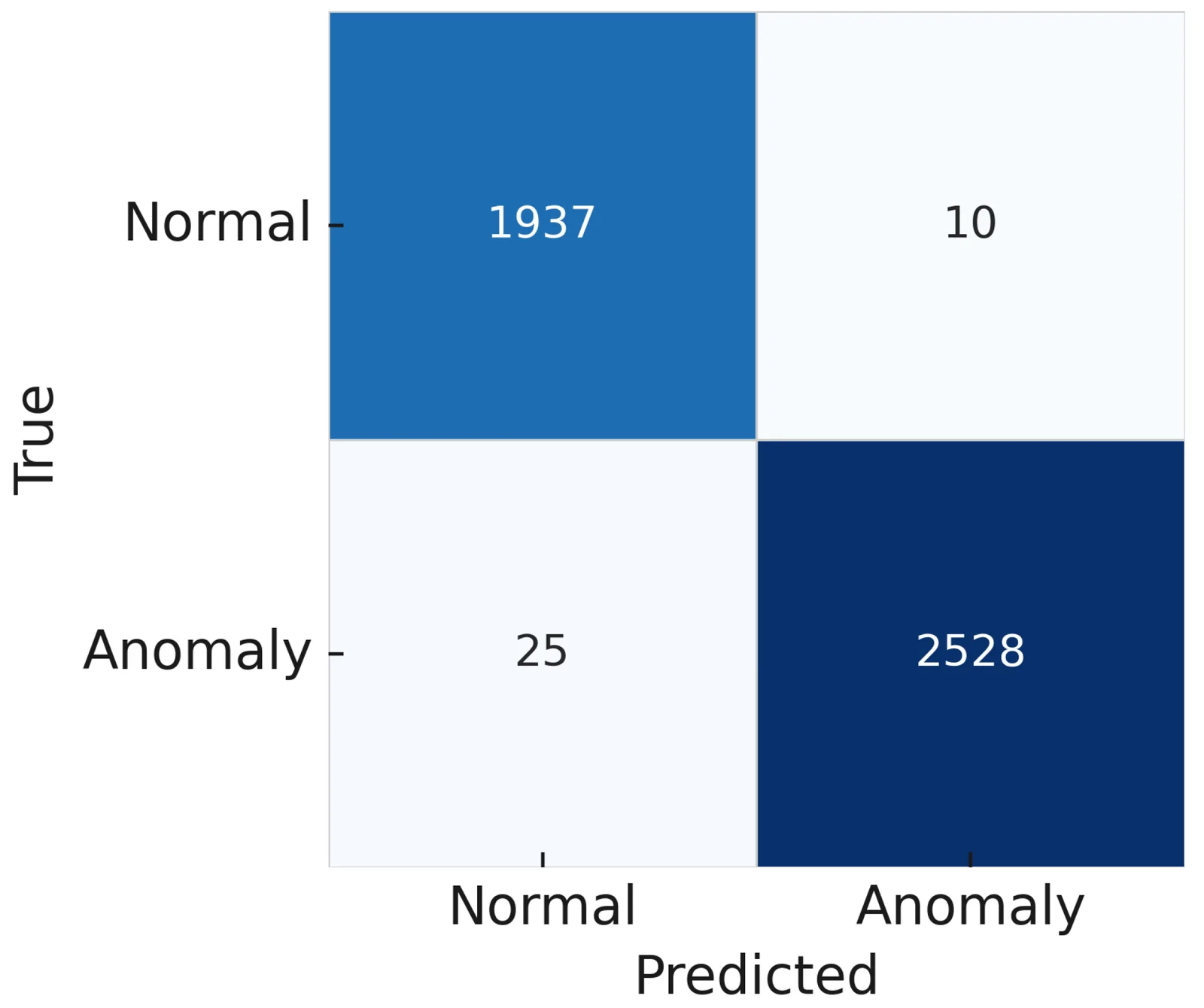
Confusion matrix of the proposed federated neuro-symbolic anomaly detection model on the held-out test set (4500 samples).

The confusion matrix in Figure 6 summarizes the held-out test classifications. Of the 1,947 normal instances, 1937 were classified correctly, while 10 were classified as anomalies. Of the 2553 anomaly instances, 2528 were correctly detected, while 25 were classified as normal. These values correspond to a normal-to-anomaly error rate of 10/1947≈0.51%, an anomaly miss rate of 25/2553≈0.98%, and an overall error rate of 35/4500≈0.78%. The results are consistent with the reported anomaly-class precision of 0.9961 and recall of 0.9902.

#### Statistical Reliability and Cross-Validation

To quantify statistical reliability, we estimated 95% confidence intervals using bootstrap resampling of the held-out test predictions. In addition, we performed five-fold patient-level cross-validation to examine whether model performance remained consistent across different train-validation partitions. Patient-level splitting was used to reduce the risk of data leakage between the training and validation folds. The slightly lower mean performance observed under five-fold cross-validation, compared with the single held-out test, indicates modest sensitivity to data partitioning. Nevertheless, the relatively small standard deviations across folds show that the model maintained broadly consistent performance under different patient-level splits. Therefore, the held-out results should be interpreted together with the cross-validation statistics rather than as isolated point estimates, providing a more balanced assessment of predictive performance in the controlled experimental setting.

As shown in Table 11, the bootstrap confidence intervals are relatively narrow for the held-out simulation results. The held-out test achieved an overall accuracy of 0.9922 and an anomaly-class F1-score of 0.9931. The five-fold patient-level cross-validation results in Table 12 show mean accuracy of 0.9844 ± 0.0044 and mean F1-score of 0.9873 ± 0.0043. Together, these results indicate that the reported performance is not based solely on a single train-test split.

### 5.3. Training Dynamics

**Figure 7:**
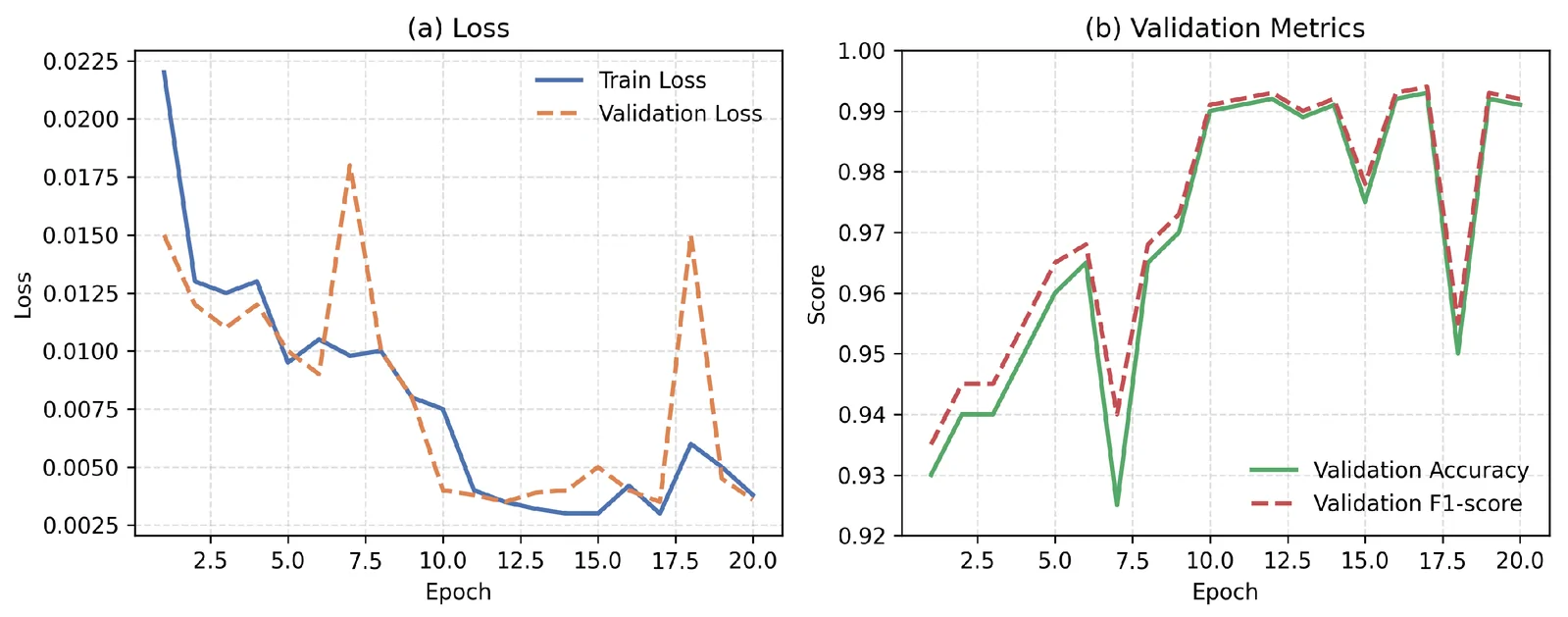
Training dynamics of the proposed model: (**a**) training and validation loss curves; (**b**) validation accuracy and F1-score across training epochs.

Figure 7 shows the training and validation curves over the training procedure. The training loss decreases rapidly and falls below 0.004 during the first 10 epochs. The validation loss follows a similar trend, with small fluctuations around epochs 7 and 17 and no sustained divergence from the training loss. The validation accuracy and F1-score exceed 0.98 after epoch 9 and reach approximately 0.993 by epoch 12, which was selected as the early-stopping checkpoint. The similar trends of the validation accuracy and F1-score indicate that both metrics improved consistently during training.

### 5.4. System-Level Simulation Results

**Figure 8:**
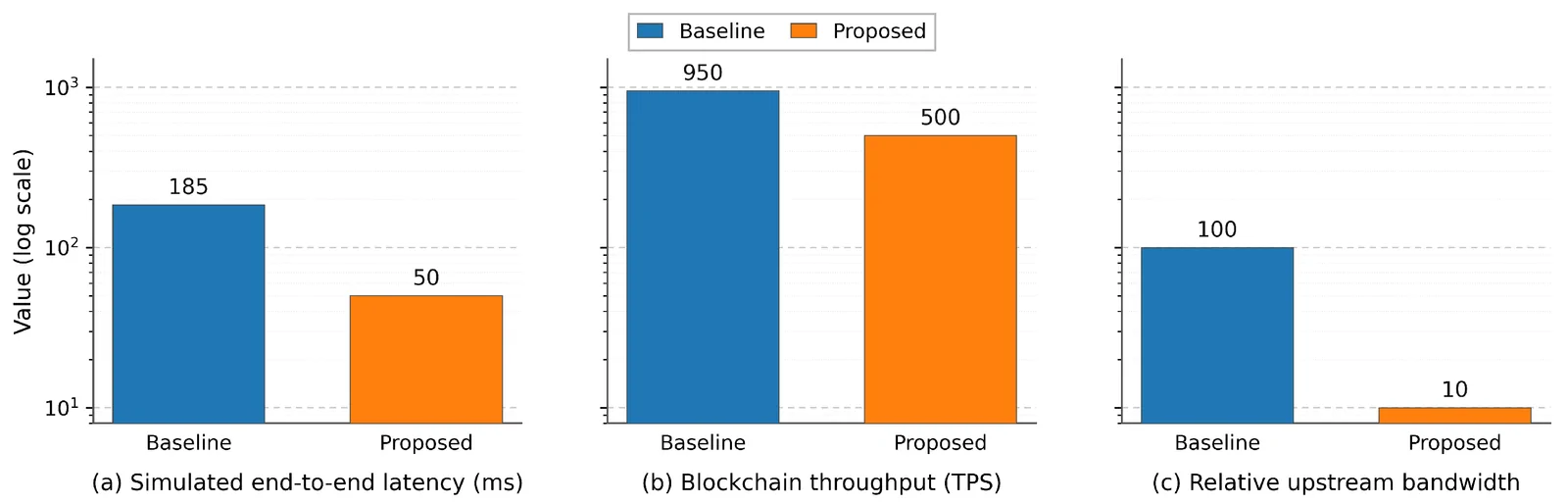
System-level results: (**a**) end-to-end latency, (**b**) blockchain throughput, and (**c**) relative upstream bandwidth.

Figure 8 summarizes three system-level indicators from the controlled simulation: end-to-end alert latency, ledger throughput, and relative upstream bandwidth. These indicators are reported in separate panels because they have different units and should not be interpreted as directly comparable values.

The end-to-end alert latency decreases from 185 ms in the baseline setting to 150 ms for cloud-only processing and to approximately 50 ms for Fog-level inference. The reduction from 185 ms to approximately 50 ms corresponds to an overall latency reduction of approximately 73%. The relative upstream bandwidth index decreases from 100 in the cloud-only setting to 10 in the Fog–Cloud setting. This reduction reflects the simulation design, in which local preprocessing retains raw data near the source and sends only processed outputs or verification metadata upstream.

The reported 50 ms value represents Fog-level inference latency for batch size 1. It should not be interpreted as verified latency from a real hospital deployment. In practice, end-to-end latency may vary according to network bandwidth, concurrent IoMT streams, Fog-node hardware, edge-device constraints, and hospital infrastructure. Therefore, real-world validation using dedicated Edge-Fog hardware remains future work. The ledger throughput is reported as approximately 500 TPS for the proposed audit-log simulation. This value is presented independently from latency and bandwidth because it represents a different system measure. Higher TPS values observed under increased transaction loads and shard configurations are reported separately in Section 5.5 as simulation-based scaling behavior rather than production–deployment measurements.

### 5.5. Blockchain Performance Evaluation

**Figure 9:**
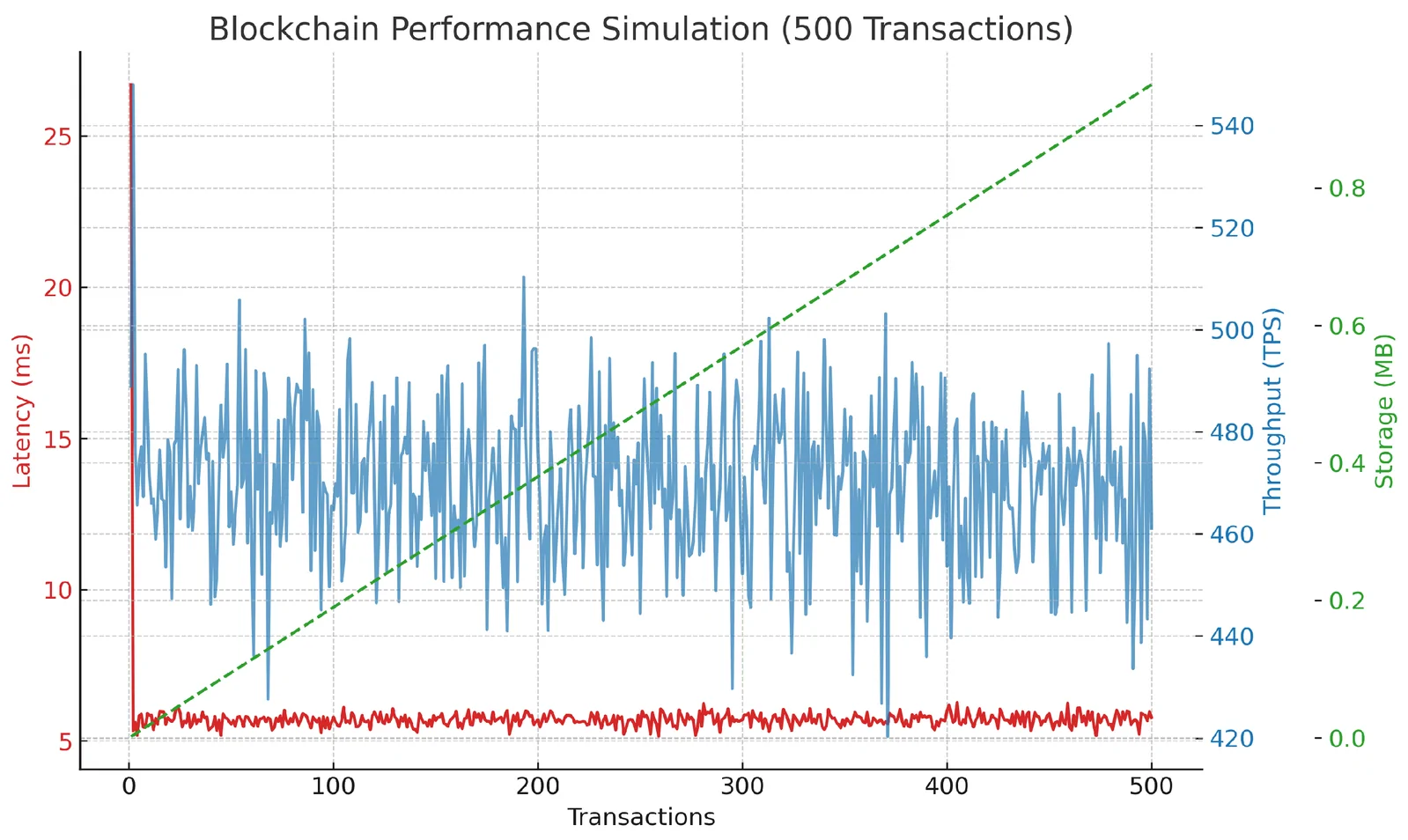
Blockchain performance during a simulation of 500 transactions. The red solid line shows transaction latency (left y-axis), the blue solid line shows throughput (inner right y-axis), and the green dashed line shows cumulative storage utilization (outer right y-axis).

The blockchain layer was first evaluated using a 500-transaction simulation to measure transaction latency, ledger throughput, and cumulative storage growth. This simulation is shown in Figure 9. The first transaction has higher latency because of initialization overhead, whereas subsequent transactions remain in an approximately 5–6 ms validation range. Throughput initially reaches approximately 548 TPS and then stabilizes around 450–500 TPS during the simulation. Cumulative storage grows approximately linearly and remains below 1 MB for 500 transactions in this controlled audit-log simulation.

**Figure 10:**
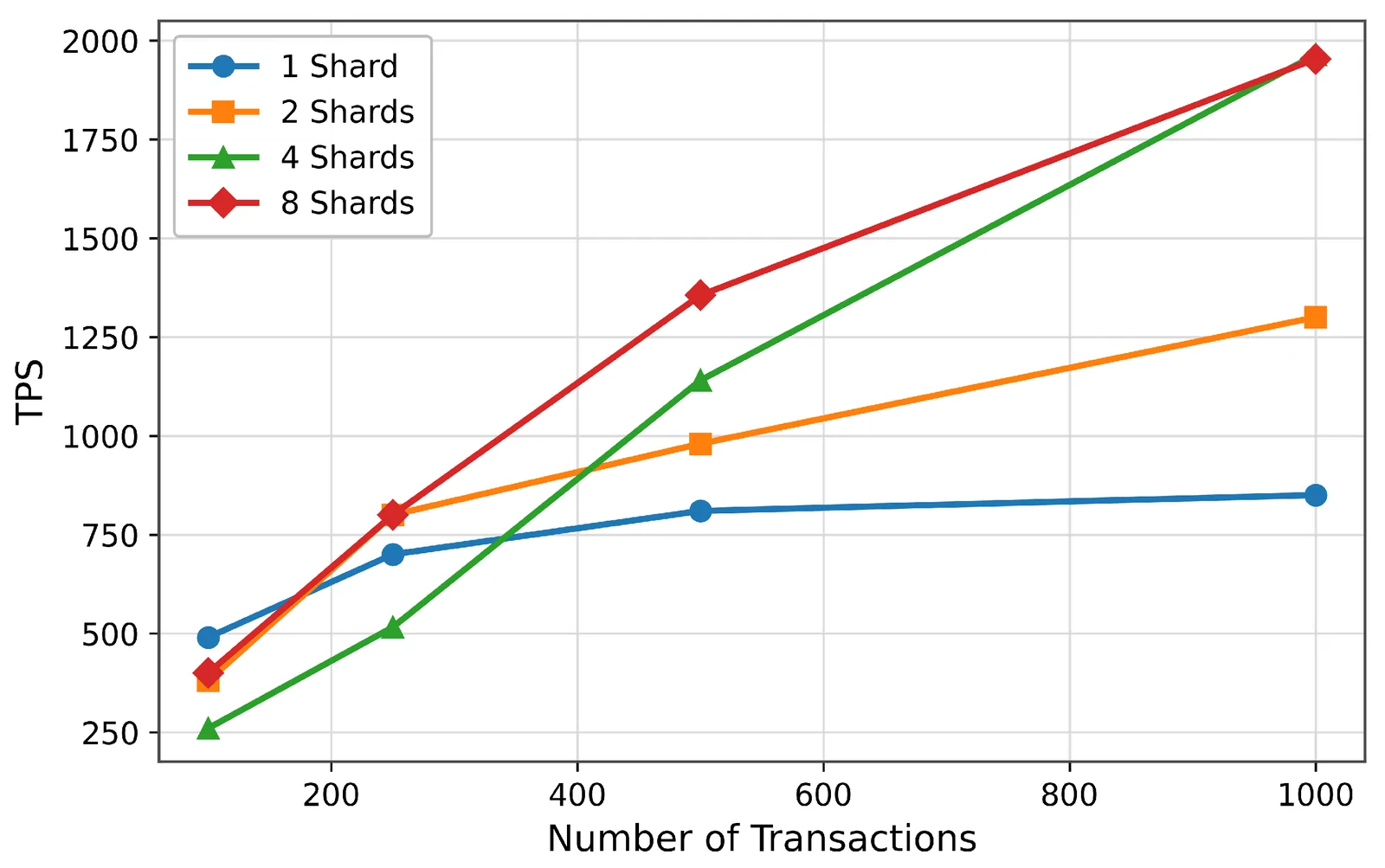
Sharded Tangle TPS scaling under different shard counts and transaction loads.

To examine scaling behavior, blockchain logging was simulated under different shard counts and transaction loads. The evaluated configurations used 1, 2, 4, and 8 shards and transaction loads of 100, 250, 500, and 1000 transactions. Transactions were assigned to shards and processed using parallel shard workers, with TPS and per-transaction latency recorded for each configuration. The results are shown in Figure 10.

In the measured setting, the ledger achieved 515.7 TPS with 4 shards and 250 transactions. At higher transaction loads, throughput reached 1140.4 TPS with 4 shards and 500 transactions and 1964.3 TPS with 4 shards and 1000 transactions. With 8 shards, throughput reached 1355.5 TPS at 500 transactions and 1953.7 TPS at 1000 transactions. These results indicate that throughput varied with transaction load and shard configuration in the controlled simulation. Higher TPS values are interpreted as simulation-based scaling behavior in larger transaction batches and shard configurations.

In addition to throughput scaling, a tamper-evident integrity-check test was performed by modifying a stored transaction hash. The audit log detected the modification through a hash mismatch, demonstrating the ability to identify this type of record alteration.

### 5.6. Comparison of FL Strategies

We evaluated several FL strategies, including FedAvg, FedProx, FedOpt, and Scaffold. The accuracy, F1-score, average training time per communication round, and GPU memory usage are summarized in Table 13. FedAvg provided a stable baseline, with 99.0% accuracy and a 99.0% F1-score. FedProx achieved the highest reported final performance, with 99.3% accuracy and a 99.3% F1-score, while requiring slightly more time per round. The proximal term in FedProx is intended to limit local-model divergence under non-IID client distributions.

Scaffold achieved a high performance range, with accuracy between 98.6% and 99.3% and F1-score between 98.8% and 99.3%. Compared with FedAvg, it required slightly more time per round and a similar amount of GPU memory. Although FedOpt provides adaptive optimization, it showed wider variation under the selected simulation configuration. Its accuracy and F1-score ranged from 72.0% to 99.2% and from 80.2% to 99.2%, respectively, indicating sensitivity to the selected learning rate and optimizer settings. In this simulation, FedProx and Scaffold provided the strongest observed accuracy-stability balance, whereas FedAvg remained a viable and computationally efficient baseline.

To further examine the role of the FL training configuration, we evaluated the sensitivity of FedProx to the local update frequency *E*. The values E∈{1,3,5,10} were tested over eight FL rounds. This short-horizon sensitivity analysis used a separate non-IID client setting; therefore, its final F1-scores are not directly comparable with the 20-round results in Table 13. The highest final F1-score, 0.8394, was obtained with E=1. Larger values of *E* produced lower and less consistent final F1-scores under the non-IID setting, as shown in Table 14.

**Figure 11:**
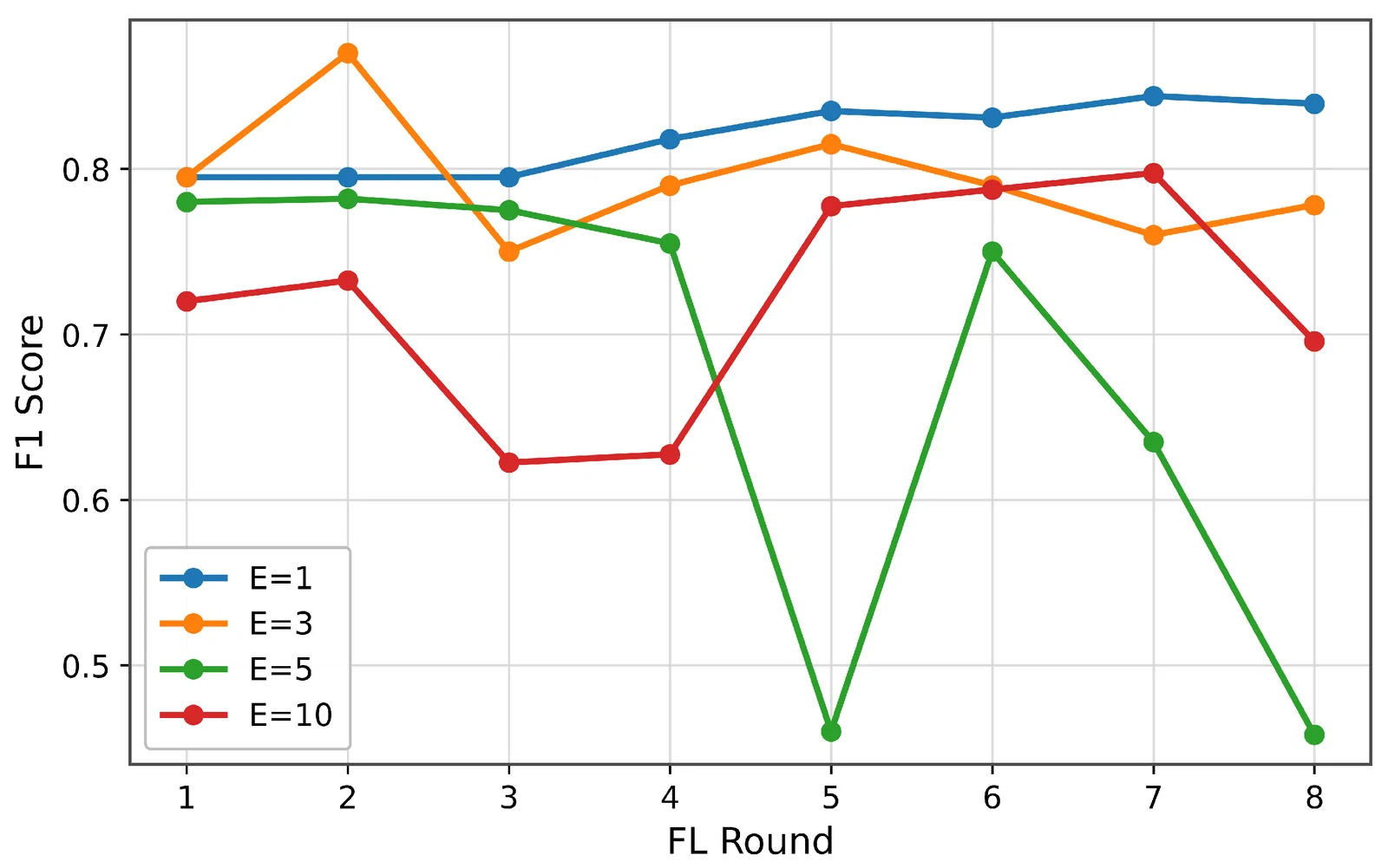
FedProx sensitivity to local update frequency *E* under the non-IID client setting. The observed results motivated the use of E=1 as the default configuration.

The results shown in Figure 11 suggest that a smaller local update frequency was associated with more stable convergence in the non-IID setting. Therefore, E=1 was selected as the default configuration for the reported FedProx experiments.

### 5.7. HomEnc Overhead Analysis

To quantify the computational and communication cost of encrypted aggregation in the controlled simulation, a CKKS microbenchmark was implemented using TenSEAL 0.3.17. The benchmark used three Fog clients, each encrypting a 500,000-element floating-point update vector. The CKKS configuration used a polynomial modulus degree of 8192 and coefficient-modulus bit sizes of [60,40,40,60]. Client-side encryption was executed sequentially in a single Colab runtime; therefore, the reported encryption time should not be interpreted as distributed wall-clock latency.

Each update was divided into 123 ciphertext chunks. The serialized payload was 38.87 MB per client, resulting in a total uplink payload of 116.62 MB for three clients, as shown in Table 15. Sequential client-side encryption required 6.74 s, while encrypted aggregation and authorized decryption required 0.13 s and 1.60 s, respectively. The maximum reconstruction error after decryption was below $10^{-5}$, confirming the functional correctness of encrypted aggregation. This component-level benchmark uses representative update vectors and does not represent end-to-end overhead for the full fine-tuned model.

### 5.8. Component-Wise Contribution

The component-level analysis distinguishes between learning components and system-security components. The learning components, including Bio-ClinicalBERT, the LSTM, attention, and symbolic regularization, are intended to affect detection performance and clinical consistency. The system-security components, including FL, HomEnc, HoneyEnc, and the sharded Tangle ledger, are intended to support data locality, encrypted aggregation, and auditability, as shown in Table 16. Therefore, not every component is expected to increase classification accuracy directly. The HomEnc component is assessed through the reported CKKS microbenchmark, whereas the ledger is assessed through the reported simulation latency, throughput, and communication analyses.

### 5.9. Explainability Analysis Using LIME Stability and Symbolic-Rule Alignment

To assess the interpretability of the proposed neuro-symbolic FL framework, we evaluated LIME-based feature attribution and symbolic-rule alignment. The model includes a Bio-ClinicalBERT attention mechanism for text fusion; however, the quantitative explainability results reported here focus on LIME stability and overlap with the symbolic-rule layer. SHAP-based multi-sample attribution and clinician-rated explanation usefulness are identified as future work.

The stability of LIME was assessed using five explanation outputs for the same anomaly sample and the variance of the feature coefficients was calculated. Lower variance indicates that similar importance values were assigned to the same features. In addition, a symbolic-rule alignment score was computed by comparing the top LIME features with the clinical rules triggered for the same sample.

Table 17 shows near-zero coefficient variance across the five explanation outputs for the selected anomaly sample. Because these values are reported to six decimal places, they should be interpreted as consistency in this limited experiment rather than as evidence of general explanation robustness. Table 18 shows an alignment score of 0.00 because no symbolic threshold rule was triggered for the selected sample. This result is reported transparently. It shows that the sample did not have direct overlap between the top LIME features and explicitly triggered rules. A broader explainability evaluation across multiple rule-triggered and non-rule-triggered anomaly samples, together with SHAP-based multi-sample attribution and clinician-rated usefulness assessment, remains an important future extension.

### 5.10. Ablation Study

To quantify the contribution of the main learning components, we performed a trained ablation study in which every model variant was trained before evaluation. The ablation study compares the full model with three trained variants: a model without Bio-ClinicalBERT, a model without the LSTM temporal encoder, and a numerical-feature-only model. As shown in Table 19, the full model achieved the best overall performance, with 99.2% accuracy and a 99.3% F1-score. Removing Bio-ClinicalBERT reduced performance to 89.6% accuracy and 91.6% F1-score, indicating that the clinical-text branch was an important contributor in the current synthetic dataset. Removing the LSTM produced 98.0% accuracy and 98.5% F1-score, indicating that temporal modeling improved the final multimodal configuration. The numerical-only model achieved 79.6% accuracy and 82.3% F1-score, showing that vital-sign values alone were less effective than the multimodal configuration in this controlled evaluation.

### 5.11. Robustness Evaluation Under Data Irregularities

To evaluate robustness under IoMT and smart-healthcare data irregularities, we tested the trained model under multiple perturbation scenarios. These scenarios included numerical sensor noise, missing vital signs with mean imputation, gradual sensor drift, reversed temporal windows, corrupted clinical text, delayed or mismatched clinical text, adversarial numerical perturbation, and class-distribution shift. These controlled scenarios approximate potential deployment problems, including sensor instability, incomplete transmissions, delayed documentation, mismatched multimodal records, and anomaly-heavy data streams.

Table 20 summarizes the results. At the reported precision, model performance changed little under the evaluated numerical perturbations, missing-vital-sign setting, sensor drift, reversed temporal windows, and FGSM perturbation. This finding should be interpreted cautiously because the synthetic labels are strongly linked to clinically inspired threshold patterns. It does not establish equivalent robustness in real clinical environments. Corrupted clinical text caused a clear performance decrease, reducing accuracy to 65.9% and F1-score to 79.4%. Delayed or mismatched text also reduced performance to 92.3% accuracy and 94.1% F1-score. These results indicate that correct multimodal alignment and the clinical-text branch are important to the model in the current synthetic evaluation.

Although the security stress tests cover model poisoning, replay attacks, false-alert injection, membership-inference proxy analysis, and Byzantine Fog-client behavior, a dedicated trigger-based backdoor-attack evaluation was not implemented in the present simulation. Therefore, resistance to backdoor attacks is not claimed as a verified result of this study. Future work will include explicit backdoor scenarios, including trigger-pattern insertion in numerical vital-sign streams and text-trigger injection in clinical notes, to evaluate whether the federated aggregation and anomaly-detection pipeline can detect or suppress persistent backdoor behavior.

### 5.12. Security Stress-Test Evaluation

To further evaluate the security behavior of the proposed framework, we performed stress-test simulations against common attacks in smart healthcare and federated IoMT environments. These attacks included model poisoning, replay attacks, false-alert injection, membership-inference proxy analysis, and Byzantine Fog-client behavior. The objective of this analysis was not to show that the security components directly improve classification accuracy, but to examine their behavior under the selected simulated attack scenarios.

Table 21 summarizes the results. Under a 10% label-flipping model-poisoning attack, model accuracy decreased by 0.5 percentage points. The replay attack was flagged through the implemented hash, timestamp, and transaction-identifier checks. When 200 fake anomaly alerts were injected, precision in that stress-test scenario decreased from 0.9993 to 0.9352, showing that false-alert injection can affect alert quality while the audit log retains event records for subsequent inspection. The membership-inference proxy test produced a confidence gap of −0.085, suggesting lower distinguishability under the evaluated setting; however, this proxy result does not constitute a formal privacy guarantee. Finally, the Byzantine Fog-client simulation produced an estimated 1–2 percentage-point F1-score decrease under the selected configuration.

These stress-test results indicate that the security components are intended to support privacy, auditability, and attack monitoring rather than directly increasing classification accuracy. FL retains raw data locally during collaborative training. The CKKS microbenchmark verifies encrypted aggregation of representative update vectors, while the sharded Tangle ledger provides tamper-evident logging and replay-validation support. HoneyEnc is included as a conceptual Fog-layer protection mechanism against brute-force recovery attempts.

### 5.13. Comparison with the State-of-the-Art

Table 22 compares the proposed framework with recent methods for secure anomaly detection in smart healthcare and IoMT settings. Centralized approaches for smart healthcare systems [25] and internet hospitals [19] report competitive detection performance. However, centralized training may increase reliance on transferring sensitive data to a central infrastructure. Alliance blockchain-based systems [19] can support integrity and auditability, but may not provide collaborative learning between distributed healthcare participants.

FL-based approaches, such as [18,29], support data locality through decentralized training. However, the reported designs do not necessarily combine multimodal clinical-text and physiological-signal fusion, neuro-symbolic learning, encrypted aggregation, Fog-level inference, and tamper-evident audit logging in one simulation framework. Hybrid AI-blockchain models for smart hospitals [1] strengthen security logging and access control but do not explicitly report a neuro-symbolic anomaly-detection design. Similarly, HomEnc-based approaches can support confidentiality during computation but may incur substantial computational overhead.

The proposed design combines multimodal neuro-symbolic learning, FL, HomEnc, Fog-level inference, and sharded Tangle audit logging. As summarized in Table 22, it achieved high held-out performance together with Fog-inference and blockchain transaction-latency results. These results should not be interpreted as a direct deployment-level comparison because the cited studies use different datasets, settings, and evaluation protocols.

### 5.14. Discussion and Practical Implications

The proposed framework is intended to support remote monitoring using wearable biosensors. Fog-based inference performs anomaly detection near the data source. This design can reduce the communication latency and the amount of raw data transmitted to the Cloud. However, the results presented here are based on synthetic data and require additional validation using real wearable devices and clinical data. Accordingly, the present findings should be interpreted as proof-of-concept results obtained from synthetic multimodal EHR-like data and controlled simulations rather than as evidence of deployment-ready clinical generalizability. External validation using real wearable biosensor data, hospital IoMT streams, or public clinical datasets such as MIMIC-III/MIMIC-IV is required before the framework can be considered for clinical deployment.

## 6. Conclusions and Future Work

Many healthcare-IoT solutions treat wearable-sensor data streams in isolation, rely on cloud-only processing, or use secure logging mechanisms without integrating multimodal learning and privacy-preserving collaborative training. This work designed and evaluated a three-tier Edge–Fog–Cloud framework that: (i) fuses numerical vital signs and clinical text through a multimodal neuro-symbolic model based on LSTM and Bio-ClinicalBERT; (ii) uses FL to preserve data locality during collaborative training and HomEnc to support encrypted aggregation; and (iii) records anomaly events through a sharded Tangle audit log to support tamper-evident traceability. In the proposed design, Fog-level inference is associated with lower end-to-end latency, while raw patient data remain local to the participating nodes. The proposed framework achieved 99.22% accuracy and a 99.31% anomaly-class F1-score, reduced end-to-end latency from 185 ms to approximately 50 ms, and achieved approximately 500 TPS. A key limitation of the present study is its evaluation using synthetic multimodal healthcare data and controlled system simulations. Therefore, the reported results should be regarded as simulation-based proof-of-concept validation rather than evidence of deployment-ready clinical generalizability. External validation using real wearable biosensor data, hospital IoMT streams, and public clinical datasets such as MIMIC-III/MIMIC-IV remains necessary. Future work will validate the framework using real wearable-biosensor datasets and hospital IoMT streams; test robustness under missing data, sensor drift, delayed documentation, and heterogeneous patient distributions; incorporate clinician feedback to refine symbolic rules and explanations; and evaluate latency, throughput, and bandwidth on dedicated Edge-Fog hardware under concurrent IoMT traffic.

## Figures and Tables

**Table 1:** Recent anomaly detection approaches in healthcare: key contributions and remaining gaps.

Ref.	Techniques/Scope	Main Limitation
[1]	Enhanced RSA, Isolation Forest, and a PoA private chain for Internet hospitals	Numerical IoT streams only; cloud-centric, with no Fog-level or multimodal fusion
[18]	Blockchain-enabled FL on EMRs with smart-contract logging	Offline EMR data; no Edge-level timing evaluation or IoT streams
[19]	Alliance blockchain with enhanced RSA for sensor data	Private chain centralizes control; transparency at scale remains unclear
[20]	IPFS and ZKP for blockchain-based EMR sharing	System-level benchmarks only; real-time latency remains unreported
[21]	PoA chain, FL, LSTM autoencoder, and smart-contract-based model management	Prototype metrics are not disclosed; deployment scalability remains untested
[22]	AFPDSM: feature-centric polynomial encryption and blockchain indexing	Encryption/decryption requires approximately 100 s per batch; high computational overhead
[23]	FL-based anomaly-detection model for hypervisor security	Simulated VM telemetry only; no heterogeneous-workload validation
[24]	Survey of identity-based, smart-contract-based, and immutable-log solutions	Scalability and interoperability remain unresolved
[25]	SHACS: role-, attribute-, and rule-based access with anomaly detection on MIMIC-III	Centralized EHR control; no blockchain integration or Fog-level enforcement
[26]	Grouped-feature autoencoders and residual learning for IoMT-blockchain traffic	No privacy-preserving training; lacks real-time Edge evaluation

**Table 2:** Physiological features and their possible wearable-sensor sources.

Physiological Feature	Possible Wearable Sensor
Heart rate	Smartwatch or wearable ECG
Blood pressure	Wearable blood-pressure monitor
Glucose	Continuous glucose monitor
Temperature	Wearable skin-temperature sensor
Respiration rate	Smart chest band
${\mathrm{SpO}}_{2}$	Wearable pulse oximeter

**Table 3:** Class distribution in the 20,000 raw training records before sequential-window construction.

Class Label	Description	Instances	Percentage
0	Normal	7463	37.3%
1	Anomaly	12,537	62.7%

**Table 4:** Prepared sequential dataset statistics after window construction.

Dataset Component	Count
Training Sequences	18,000
Training Labels	18,000
Training Clinical Texts	18,000
Testing Sequences	4500
Testing Labels	4500
Testing Clinical Texts	4500

**Table 5:** Clinically inspired threshold rules used in the neuro-symbolic constraint layer.

Rule	Vital-Sign Condition	Interpretation	Rule Direction
$R_{1}$	HR>100 bpm	Tachycardia	Upper-bound violation
$R_{2}$	HR<60 bpm	Bradycardia	Lower-bound violation
$R_{3}$	$BP_{\mathrm{sys}}>140$ mmHg	Hypertension	Upper-bound violation
$R_{4}$	$BP_{\mathrm{sys}}<90$ mmHg	Hypotension	Lower-bound violation
$R_{5}$	Glucose>180 mg/dL	Hyperglycemia	Upper-bound violation
$R_{6}$	Glucose<70 mg/dL	Hypoglycemia	Lower-bound violation
$R_{7}$	Temp>38 °C	Fever	Upper-bound violation
$R_{8}$	${\mathrm{SpO}}_{2}<94\%$	Hypoxia	Lower-bound violation

**Table 6:** Comparison of audit-log alternatives for smart healthcare deployment.

Approach	Strengths	Limitations	Suitability for the Proposed Framework
Secure append-only database	Low latency and simple deployment; efficient for single-hospital logging	Trust is usually centralized; limited cross-site auditability and independent verification	Suitable for small single-site deployments, but less suitable for distributed Fog-level trust verification
Permissioned blockchain	Shared governance, access control, and tamper-evident records	Block ordering and consensus may create throughput and latency overhead under high IoMT event rates	Suitable for regulated consortium settings, but heavier for frequent anomaly-event logging
Non-sharded DAG ledger	Asynchronous transaction attachment and lightweight event recording	Less explicit workload partitioning when many Fog nodes generate concurrent logs	Suitable for moderate event rates, but less scalable than explicit sharding
Proposed sharded Tangle ledger	Parallel audit logging, tamper-evident records, and workload distribution across shards	Requires shard-management logic and careful deployment configuration	Designed for multi-Fog or multi-institution IoMT settings with frequent anomaly and model-update logs

**Table 7:** Qualitative comparison with mainstream blockchain deployment categories.

Blockchain Category	Main Strength	Limitation for Smart Healthcare Audit Logging	Suitability
Ethereum-style public blockchain	Strong decentralization and public verifiability	Public execution environment, transaction cost, privacy concerns, and latency may reduce suitability for sensitive healthcare audit logs	Low
Hyperledger/Fabric-style permissioned blockchain	Access control, consortium governance, and enterprise-deployment support	Ordering and consensus overhead may increase when many Fog nodes generate frequent anomaly and model-update records	Moderate to high
Non-sharded DAG/IOTA-style ledger	Asynchronous transaction attachment and lightweight event recording	Without explicit sharding, larger-scale concurrent logging may create workload-management challenges	Moderate
Secure append-only database	Simple deployment, low overhead, and efficient single-site logging	Centralized trust model and limited cross-institution auditability	Moderate for single-site use
Proposed sharded Tangle ledger	Parallel audit logging, tamper-evident records, and workload distribution across shards	Requires shard-routing logic and real-world deployment validation	Potentially suitable for the multi-Fog setting

**Table 8:** Software stack, model hyperparameters, and blockchain settings used in the controlled experiments.

Key Implementation Parameters	
**Library/Tool**	**Version/Setting**
Python	3.10 (Google Colab Pro)
PyTorch (CUDA)	1.13 with CUDA 11.7
Transformers	4.33
Bio-ClinicalBERT model	emilyalsentzer/Bio_ClinicalBERT
Flower FL framework	1.7
TenSEAL (CKKS)	0.3.17 (poly. modulus degree 8192; coefficient-modulus bit sizes [60,40,40,60])
Cryptographic tools	cryptography 41.0 and Python hashlib module
Pandas/NumPy	1.5/1.24
Ledger evaluation	Python-based sharded Tangle audit-log simulation
**Hyperparameter**	**Value**
Sliding-window length *T*	5 records
Maximum text length	256 tokens
LSTM hidden size	64
Dropout rate	0.30
Optimizer	AdamW, learning rate $1\times 10^{-4}$, weight decay $1\times 10^{-5}$
Loss function	Focal loss (α=0.25, γ=2.0)
Batch size	128
FL training rounds	20
Tangle configuration	4 shards

**Table 9:** Controlled simulation setup and measurement assumptions.

Measurement Aspect	Setting/Interpretation
Execution environment	Google Colab Pro using Python 3.10 and an NVIDIA T4 GPU with 16 GB of memory
Fog clients	Fog clients implemented as separate workers in the controlled runtime environment
Cloud coordinator	Separate simulation process for global-model aggregation, HomEnc aggregation logic, and ledger validation
Dataset setting	Synthetic multimodal EHR-like dataset with temporal vital signs and aligned clinical-text entries
Inference latency	Fog-level inference latency recorded for local anomaly detection
Blockchain throughput	Measured using a Python-based sharded Tangle audit-log simulation with shard workers
Shard settings	1, 2, 4, and 8 shards
Transaction loads	100, 250, 500, and 1000 transactions
TPS value	Approximately 500 TPS in the simulation setting
Higher TPS values	Shard-scaling simulation behavior under increased transaction loads and shard counts
Bandwidth metric	Relative upstream bandwidth index rather than physical network bandwidth measured in Mbps

**Table 10:** Per-class and aggregate performance on the held-out test set.

Class	Precision	Recall	F1-Score	Support
Normal (0)	0.9873	0.9949	0.9910	1947
Anomaly (1)	0.9961	0.9902	0.9931	2553
Accuracy	0.9922			4500
Macro average	0.9917	0.9925	0.9921	4500
Weighted average	0.9923	0.9922	0.9922	4500

**Table 11:** Bootstrap 95% confidence intervals for held-out test performance.

Metric	Mean	Lower 95% CI	Upper 95% CI
Accuracy	0.9922	0.9896	0.9947
Precision	0.9961	0.9934	0.9984
Recall	0.9902	0.9862	0.9938
F1-score	0.9931	0.9908	0.9953

**Table 12:** Five-fold patient-level cross-validation results.

Fold	Accuracy	Precision	Recall	F1-Score
1	0.9858	0.9960	0.9829	0.9894
2	0.9782	0.9977	0.9663	0.9818
3	0.9882	0.9955	0.9864	0.9910
4	0.9816	0.9944	0.9730	0.9836
5	0.9882	0.9971	0.9840	0.9905
Mean ± Std.	0.9844 ± 0.0044	0.9962 ± 0.0013	0.9785 ± 0.0085	0.9873 ± 0.0043

**Table 13:** Comparison of FL strategies under the Fog-client setting.

FL Strategy	Accuracy (%)	F1-Score (%)	Avg. Time/Round (s)	GPU Usage (MB)
FedAvg	99.0	99.0	82.1	5377
FedProx	99.3	99.3	83.6	5385
FedOpt	72.0–99.2	80.2–99.2	82.0	5773
Scaffold	98.6–99.3	98.8–99.3	83.2	5387

Note: Accuracy and F1-score are reported as percentages. Average training time is reported per communication round, and GPU usage is reported in MB. FedAvg and FedProx values are final results, whereas FedOpt and Scaffold values indicate the range observed across FL rounds.

**Table 14:** Sensitivity analysis of FedProx local update frequency *E* under non-IID client distributions.

Local Update Frequency *E*	Final F1-Score
1	0.8394
3	0.7782
5	0.4579
10	0.6957

**Table 15:** Measured CKKS encrypted-aggregation overhead in the controlled microbenchmark using three Fog clients and 500,000-element update vectors.

Measurement	Value
Ciphertext chunks per update	123
Serialized payload per client (MB)	38.87
Total serialized uplink (MB)	116.62
Sequential client-side encryption time (s)	6.74
Encrypted aggregation time (s)	0.13
Authorized decryption time (s)	1.60
Maximum reconstruction error	<$10^{-5}$

**Table 16:** Component-wise contribution of the proposed framework.

Component	Main Contribution	Evidence in Manuscript
LSTM temporal encoder	Models temporal dependencies in vital-sign sequences	Removing the LSTM reduced performance from 99.22% accuracy and 99.31% F1-score to 98.0% accuracy and 98.5% F1-score.
Bio-ClinicalBERT	Extracts contextual representations from clinical text	Removing Bio-ClinicalBERT reduced performance to 89.6% accuracy and 91.6% F1-score, indicating the importance of the clinical-text branch in the synthetic evaluation.
Attention mechanism	Weights text-token representations during multimodal fusion	Included in the text-fusion branch; LIME stability analysis is reported in Section 5.9.
Symbolic regularization	Encourages consistency with clinically inspired vital-sign rules	Integrated into the training objective and supported by the clinical-rule formulation and robustness analysis.
FL	Enables collaborative model training without centralizing raw patient data	Evaluated through FL strategy comparison and *E*-sensitivity analysis. FedProx with E=1 showed the most stable observed behavior under the non-IID setting.
HomEnc	Supports encrypted aggregation in the FL design	Evaluated through a CKKS microbenchmark using three Fog clients and 500,000-element update vectors; the serialized payload was 38.87 MB per client.
HoneyEnc	Provides conceptual Fog-layer protection against brute-force recovery attempts	Contributes to security design rather than classification accuracy; quantitative HoneyEnc evaluation remains future work.
Sharded Tangle ledger	Supports tamper-evident audit logging, replay checks, and event traceability	Evaluated through throughput scaling and a hash-integrity check. The simulation detected a modified stored hash under the tested configuration.

**Table 17:** LIME explanation stability for the selected anomaly sample.

Feature	Mean Coefficient	Variance	Stable
HR	−0.0005	0.000000	Yes
${\mathrm{BP}}_{\mathrm{sys}}$	0.0000	0.000000	Yes
${\mathrm{BP}}_{\mathrm{dia}}$	0.0008	0.000000	Yes
Glucose	−0.0021	0.000000	Yes
Temp	0.0003	0.000000	Yes
RespRate	0.0010	0.000000	Yes
${\mathrm{SpO}}_{2}$	−0.0001	0.000000	Yes
Mean variance	0.000000		

**Table 18:** LIME/symbolic-rule alignment for the evaluated anomaly sample.

Item	Result
Triggered symbolic rules	None for the selected sample
Top LIME features	Glucose, RespRate, ${\mathrm{BP}}_{\mathrm{dia}}$
Alignment score	0.00
Interpretation	The selected sample did not activate an explicit threshold-based symbolic rule; therefore, rule overlap was not observed for this sample.

**Table 19:** Trained ablation study showing the contribution of core learning components.

Model Variant	Accuracy (%)	F1-Score (%)
Full model (Bio-ClinicalBERT + LSTM + symbolic regularization)	99.2	99.3
No Bio-ClinicalBERT (LSTM + numerical features)	89.6	91.6
No LSTM (Bio-ClinicalBERT + numerical features)	98.0	98.5
Numerical features only (MLP)	79.6	82.3

**Table 20:** Robustness evaluation under numerical perturbations, text corruption, adversarial perturbation, and class-distribution shift.

Scenario	Accuracy (%)	F1-Score (%)
Clean baseline	99.2	99.3
Gaussian noise (10% feature standard deviation)	99.2	99.3
30% missing vital signs (mean imputation)	99.2	99.3
Sensor drift (gradual 25% standard deviation)	99.2	99.3
Reversed temporal window	99.2	99.3
Corrupted clinical text	65.9	79.4
Delayed/mismatched clinical text	92.3	94.1
FGSM adversarial perturbation (10% feature scale)	99.2	99.3
Class-distribution shift (80% anomaly)	98.6	99.1

**Table 21:** Security stress-test results under the controlled simulation setting.

Attack Scenario	Observed Impact	Relevant Framework Mechanism
Model poisoning with 10% label flipping	0.5-percentage-point accuracy decrease	FedProx, class weighting, and gradient clipping in the evaluated configuration
Replay attack	Flagged by hash, timestamp, and transaction-identifier checks	Sharded Tangle audit logging and transaction-validation checks
False-alert injection with 200 fake records	Precision decreased from 0.9993 to 0.9352	Audit-log traceability for alert records
Membership-inference proxy	Confidence gap of −0.085	FL data locality and encrypted-aggregation design
Byzantine Fog client	Estimated 1-2-percentage-point F1-score decrease	FedProx proximal regularization and gradient clipping in the evaluated configuration

**Table 22:** Comparison with recent state-of-the-art methods.

Study	Application/Detection Task	Learning and Security Mechanism	Reported Performance/Overhead
[25]	Smart healthcare; healthcare anomaly detection	Centralized deep learning; secure access control	Accuracy ≈97.8%; F1 ≈0.97
[19]	Internet hospitals; abnormal behavior detection	Centralized Machine Learning; alliance blockchain (PoA)	Accuracy ≈96.4%; blockchain latency ≈12–15 ms
[18]	IoMT networks; intrusion/anomaly detection	FL; blockchain-secured FL aggregation	Accuracy ≈98.6%; F1 ≈0.98
[1]	Smart hospitals; network and access anomalies	Hybrid AI models; blockchain-based secure logging	Accuracy ≈98.1%
[29]	IoT and edge systems; IoT anomaly detection	Hierarchical FL; blockchain consensus	Accuracy ≈98.9%; F1 ≈0.989
[30]	Secure IoT systems; encrypted anomaly detection	Centralized deep learning; HomEnc	Accuracy ≈97.2%; high cryptographic overhead
This work	IoMT and smart healthcare; healthcare anomaly detection	Federated multimodal neuro-symbolic learning; FL, HomEnc, and sharded Tangle audit logging	Accuracy =99.2%; anomaly F1 =0.993; Fog-inference latency ≈50 ms; blockchain transaction latency ≈5–6 ms; simulation throughput ≈500 TPS

## Data Availability

The synthetic multimodal EHR-like data used in this study were generated solely for controlled simulations. No real patient data or external clinical dataset was used.
